# Heterogeneous responses to low level death receptor activation are explained by random molecular assembly of the Caspase-8 activation platform

**DOI:** 10.1371/journal.pcbi.1007374

**Published:** 2019-09-25

**Authors:** Anna Matveeva, Michael Fichtner, Katherine McAllister, Christopher McCann, Marc Sturrock, Daniel B. Longley, Jochen H. M. Prehn

**Affiliations:** 1 Centre for Systems Medicine, Royal College of Surgeons in Ireland, Dublin, Ireland; 2 Department of Physiology and Medical Physics, Royal College of Surgeons in Ireland, Dublin, Ireland; 3 Centre for Cancer Research and Cell Biology, Queen’s University, Belfast, United Kingdom; National Institutes of Health, UNITED STATES

## Abstract

Ligand binding to death receptors activates apoptosis in cancer cells. Stimulation of death receptors results in the formation of intracellular multiprotein platforms that either activate the apoptotic initiator Caspase-8 to trigger cell death, or signal through kinases to initiate inflammatory and cell survival signalling. Two of these platforms, the Death-Inducing Signalling Complex (DISC) and the RIPoptosome, also initiate necroptosis by building filamentous scaffolds that lead to the activation of mixed lineage kinase domain-like pseudokinase. To explain cell decision making downstream of death receptor activation, we developed a semi-stochastic model of DISC/RIPoptosome formation. The model is a hybrid of a direct Gillespie stochastic simulation algorithm for slow assembly of the RIPoptosome and a deterministic model of downstream caspase activation. The model explains how alterations in the level of death receptor-ligand complexes, their clustering properties and intrinsic molecular fluctuations in RIPoptosome assembly drive heterogeneous dynamics of Caspase-8 activation. The model highlights how kinetic proofreading leads to heterogeneous cell responses and results in fractional cell killing at low levels of receptor stimulation. It reveals that the noise in Caspase-8 activation—exclusively caused by the stochastic molecular assembly of the DISC/RIPoptosome platform—has a key function in extrinsic apoptotic stimuli recognition.

## Author summary

Death receptors are targets of novel cancer therapeutics. Most of them signal through flexible multiprotein platforms to either activate apoptotic or necroptotic cell death, or propagate cell survival and pro-inflammatory signals. We focused our study on the role of dynamic assembly and composition of these platforms in the initiation of cell death at the single cell level. Since the assembly is slow through the competitive nature of protein binding within the platforms core we developed a stochastic mathematical model of the death inducing signalling platform. Our model provided an explanation for delayed cell death and fractional killing upon the death receptor stimulation. Additionally, we found that the variability in the cell death response arises through the random assembly initiates a slow noise-prone ramp activation of initiator Caspase-8 spontaneously triggering the apoptotic cascade. Our computational simulations predicted high variation in the time required for cell death induction at the single cell level and highlighted a significant role of death receptor clustering in effective Caspase-8 activation. Our knowledge and data driven model captures detailed processes governing the early events of cell death initiation and can be used to guide the development of more rational combinational treatments against cancer.

## Introduction

Apoptotic signalling cascades are designed to irreversibly lead to cell death once specific death thresholds are overcome [1,2]. Activation of caspases plays a central role in this process. In certain scenarios, apoptotic cell death signalling is interrupted. This may lead to the activation of other forms of cell death or escape from cell death altogether.

**Fig 1:**
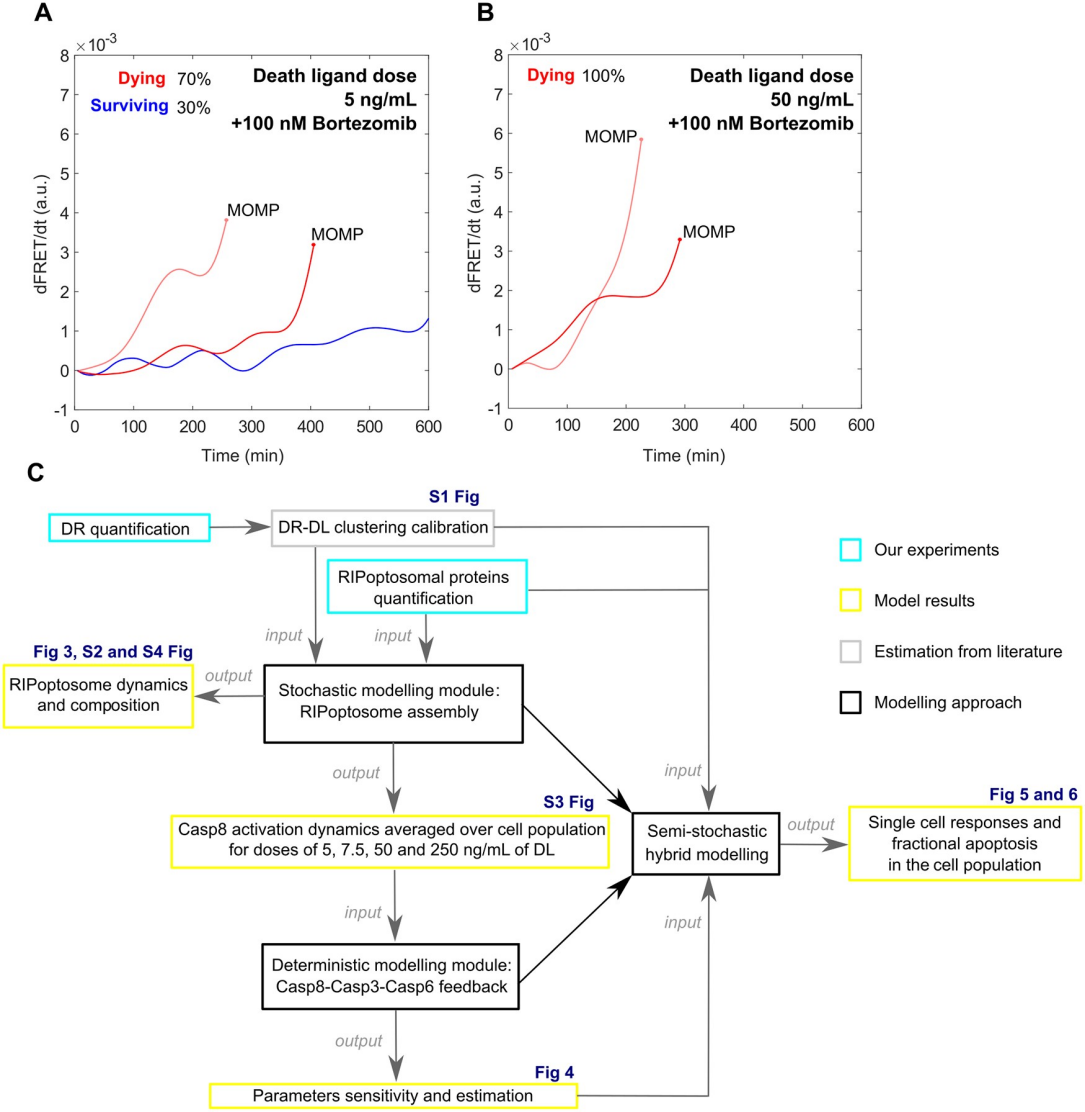
New modelling approach developed in this study to explain vulnerable dynamics of Casp8 activation and fractional cell death upon DR stimulation. (A, B) Casp8 activation dynamics tracked by the FRET reporter cleavage in Hela cells upon treatment with low and high doses of the DR ligand (experimental data from Roux *et al*., 2015)[2] as evidence for stochastic fluctuation dynamics and final switch like transition of the Casp8 activity which is the main cause of the fractional cell death. (A) Single cell Casp8 activation upon 5 ng/mL rhTRAIL treatment with 100 nM Bortezomib. Fluctuating ramp in Casp8 activation which leads to activity acceleration and MOMP within first 10 hours (in red) and fluctuating ramp which fails to trigger MOMP (in blue) causing survival of 30% cell population (experimental data from Roux *et al*., 2015) [2]. (B) 100% cell death over population induced by 50 ng/mL rhTRAIL with 100 nM Bortezomib treatment triggered by high acceleration rate of Casp8 activity (in red). (C) Schematic diagram representing the methodological design of this study.

Death ligands (DL) bind to death receptors (DR) at the plasma membrane and have been developed as novel cancer therapeutics. However, many cells in our body are exposed from time to time to endogenous DLs, such as TNF-α and TRAIL, without induction of cell death. Several studies have shown that while binding of DLs to DRs can induce apoptosis, not all cells will respond to DR stimulation with cell death, and only a fraction of the cell population will undergo apoptosis even if DLs bind at death-inducing concentrations [2–6] (Fig 1A). Interestingly, *in vivo* studies have shown that fractional death resistance has no direct association with the amount of DRs expressed on the plasma membrane [7,8]. Therefore, cell signalling activated by extrinsic ‘death’ signals is rather encoded downstream of receptor binding.

Binding of DLs to dedicated DRs triggers either the formation of receptor-associated Death-Inducing Signalling Complexes (DISC) (‘Complex I’) in proximity to the plasma membrane, or RIPoptosome complexes (‘Complex II’) in the cytosol [5,9–17]. Both complexes provide a platform for the activation of the initiator Caspase-8 (Casp8). For the activation of Casp8, the inactive pro-form of Casp8 (ProCasp8) must undergo autocatalytic activation. This is achieved through ProCasp8 dimerization and sequential inter- and intradimer cleavage, a process which results in the release of active Casp8 [18–20]. The dimeric ProCasp8 association-dissociation balance has been suggested to play a crucial role in the molecular control of apoptotic responses after DR activation [21]. However, as demonstrated by mutagenesis studies, ProCasp8 dimerization alone is not sufficient to enhance apoptotic responses *in vivo* [22]. Instead, formation of the DISC or RIPoptosome platforms are necessary for effective ProCasp8 dimerization and Casp8 activation [10,23,24].

**Fig 2:**
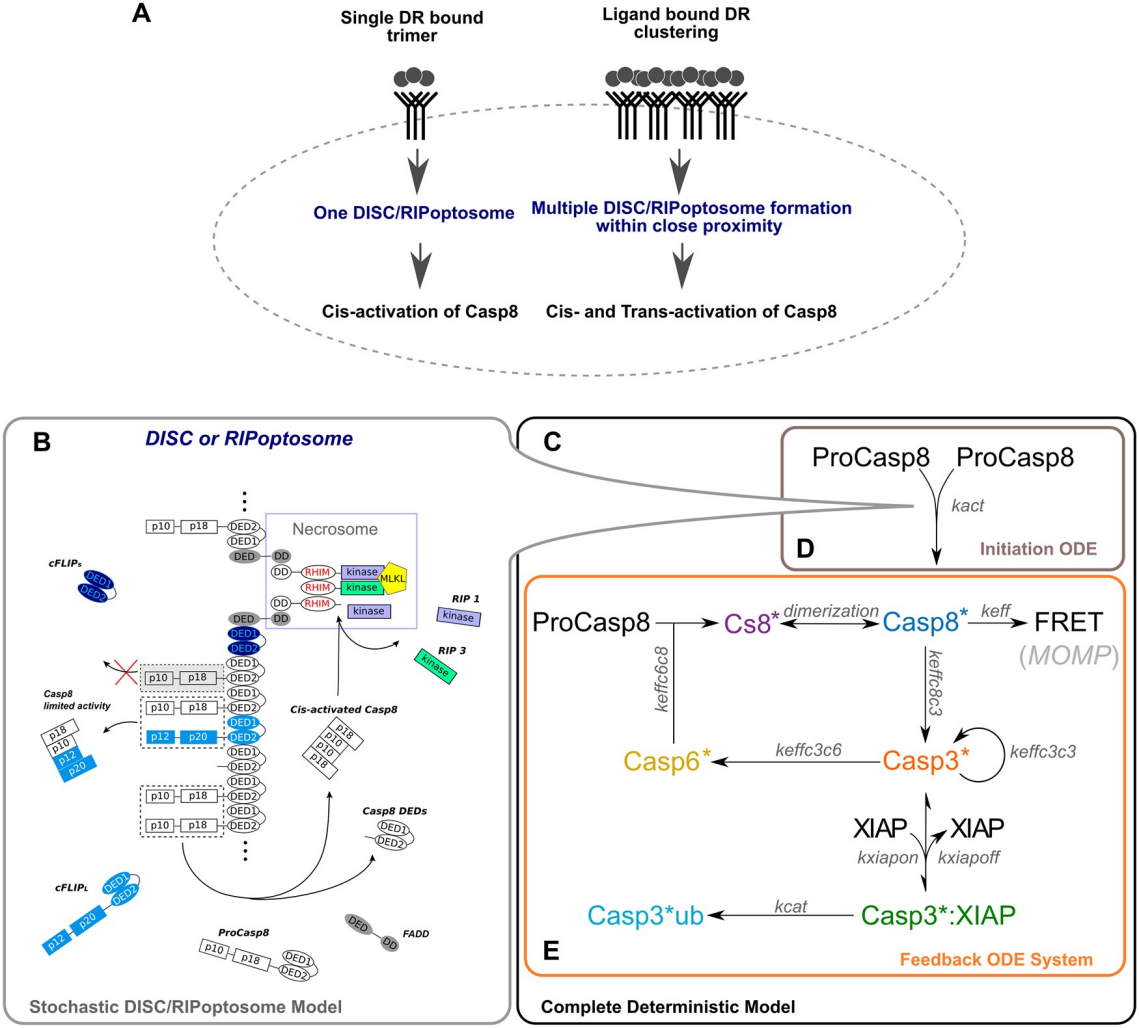
New modelling approach developed in this study to explain vulnerable dynamics of Casp8 activation and fractional cell death upon DR stimulation. (A) The DR clustering initiates the formation of a few RIPoptosomes in the closed proximity to each other where Casp8, in addition to cis-activation, can undergo trans-activation. (B) The initial stochastic Casp8 activation on the DISC/RIPoptosome platform is implemented by direct Gillespie stochastic simulation algorithm (SSA) which incorporates the molecular assembly of FADD, RIP1, RIP3, ProCasp8, cFLIPs/l proteins. (C) Complete deterministic system comprised of initiation ODE system (D) and feedback ODE system (E) used in the parameter scan and manual parameter adjustment. (D) Box represents the deterministic approximation of the population kinetics of Casp8 activation on the DISC/RIPoptosome platform. (E) Box represents the deterministic activation of the effector caspases, Casp3 and Casp6, which feedback to the rate of Casp8 activation before MOMP.

Apart from apoptosis initiation, DR-induced complexes also initiate necroptosis by accumulating heterodimers of receptor-interacting proteins (RIPs), RIP1 and RIP3 (RIP1/3), and the formation of filamentous scaffolds [25–28]. Formation of such ‘Necrosome’ platforms activates the mixed lineage kinase domain-like (MLKL) pseudokinase. MLKL activation triggers necroptosis, a cell death distinct from apoptosis [29–31]. In theory, activation of DRs in individual cells could lead to both apoptosis and necroptosis signalling through the formation of different platforms. However, if RIP1/3 proteins are close to the site of Casp8 activation, RIP1/3 is cleaved by Casp8 [32]. This cleavage eliminates the kinase activity of RIP1/3, and consequently necroptosis activation is suppressed [9,33–36] (Fig 2B). This suggests that if one type of cell death is triggered in a given cell, the other type of cell death is suppressed, i.e., that the two types of cell death are mutually exclusive.

Previous studies of the apoptotic signalling network activated by DRs have identified that variability in death signalling arises from the process preceding the mitochondrial outer membrane permeabilization (MOMP). This process triggers Casp8-mediated cleavage of the pro-apoptotic Bid protein [2,4,37], which mediates MOMP and leads to cytochrome-C release, apoptosome formation and executioner caspase activation [38].

To understand cell death decision making in more detail, we created a mathematical model which incorporates the central events prior to Bid cleavage. The model was constructed to estimate apoptotic and necroptotic pathway initiation through the random assembly of the DISC/RIPoptosome platform. As a multiprotein platform with diverse functionality, we hypothesised that the random and stochastic process of its assembly may lead to the heterogeneous cellular responses (Fig 1A and 1B). Combining this model with experimentally derived sets of quantitative protein profiles and literature-based catalytic and binding rates, we simulated the heterogeneous responses of HeLa cells to DR activation. By modelling different conditions of DR stimulation and clustering, we investigated in particular how heterogeneous apoptotic responses arise, which role the random assembly of DR-induced platforms play in determining death delay at the single cell level, and how DR clustering facilitates death signalling. Our analysis reveals that the noise in Casp8 activation exclusively caused by the stochastic molecular assembly of the DISC/RIPoptosome platform has a key function in the low level extrinsic apoptotic stimuli recognition.

## Results

### Quantitative estimation of death receptor abundance and clustering

Apoptosis inducing DRs such as Tumour Necrosis Factor Receptor 1 (TNFR1) and Death Receptors 4 and 5 (DR4/5) are expressed at comparable protein levels in HeLa cells [39]. Additionally, it is known that their protein expression level is correlated with the receptor abundance on the cell surface [8]. High variation in TNFR1 surface abundance were estimated in previous studies ranging from 300 to 3000 molecules per single HeLa cell [40,41]. To get more accurate estimates, we performed the single cell quantification of TNFR1 membrane expression in HeLa cells employing the QuantiBRITE phycoerythrin beads based assay (see S1 File). We determined that the average number of TNFR1 does not exceed 905 receptors per cell. We further used this quantity as the reference in our comparative quantification of DR4/5 receptors based on MS data set (S1 File). Thus, we calculated that DR4 and DR5 receptors are present on HeLa cell surface in an average amount of 769 and 926 monomeric receptors, respectively (Table A in S1 File).

Next, we estimated the amount of the DR complexes associated with DL at the single cell level. Due to the fact that the DR-DL association is generally much quicker [42] than the downstream processes such as ProCasp8 dimerization and subsequent Casp8 activation [43], we applied the rapid equilibria approximation to calculate the amount of DL bound receptors. According to the law of mass action the time evolution of the amount of DR-DL complexes is

$$\frac{d[RL]}{dt}=k_{on}[R][L]-k_{off}[RL],\;where\;[R]=[R_{total}]-[RL]$$ (1)

Where [*R*_*total*_] is the total number of receptors per cell (Table A in S1 File), [*RL*] is the number of DR-DL complexes and [*L*] is death ligand concentration (Table B in S1 File).

Setting the RL to the rapid equilibrium

$$\frac{d[RL]}{dt}=0$$ (2)

From (1) we calculated the average number of DR-DL complexes per cell as a function of *L*, *R*_*total*_ and the DL dissociation constant *K*_*d*_

$$[RL]=\frac{{[R}_{total}]}{\left(\frac{K_{d}}{\left[L\right]}+1\right)}$$ (3)

The minimal unit of the active DR-DL complex is the trimer [44]. The trimeric DR-DL complex gives birth to a single DISC platform which internalizes within the subsequent 10–15 minutes [45,46]. If the DISC has not bound to cellular Inhibitor of Apoptosis Proteins (cIAPs), cIAP1 or cIAP2, then it either releases active RIP1 protein into the cytosol [47] where it can form RIPoptosome or Necrosome platforms (Fig 2B) (as in case of TNFR1), or it makes active RIP1 protein accessible for further RIP1/3 and ProCasp8 proteins accumulation on the DISC itself (as in case of DR4/5 activation) [5,17]. Therefore, in the modelling routine each activated DISC was translated into a single RIP1 protein molecule which is available immediately after DL introduction to the cell culture.

Trimeric DR-DL complexes tend to organise high order clusters in cellular membranes [44,48] and bring several associated DISC/RIPoptosomes into close proximity. Such clustering stimulates more efficient signalling [49] and enables ProCasp8 activation not only by dimerization on the single DISC/RIPoptosome but also by synchronised binding of two ProCasp8 monomers with two independent DISC/RIPoptosomes within one cluster. To introduce DISC/RIPoptosomes clustering processes in the model, we estimated the number and the size of the DR-DL clusters based on the experimentally derived DR-DL probability distribution from a study published earlier by Fricke and co-workers [44]. We calibrated probability redistribution from the total pool of activated trimeric DR-DL complexes, calculated in the previous step, to the clusters of different size (see S1 File). Using these probabilities, we assigned for each random DISC/RIPoptosomes complex formed its associated cluster. The final algorithm assumes that DISC/RIPoptosomes complexes within one cluster are able to first encourage the activation of ProCasp8 by direct dimerization (cis-activation) and subsequently activate ProCasp8 via simultaneous binding within closed proximity (trans-activation) (Fig 2A). Thus, this information about the amount of the activated DR-DL complexes and their clustering conformation served as an important input for the model. The scenario of non-clustering DR signalling was studied as well by setting the probability of trans-activation of Casp8 within DISC/RIPoptosomes complexes cluster to zero. This scenario is hereafter referred to as disrupted clustering.

### Mathematical modelling of the initiation of the extrinsic apoptosis pathway

We have developed a core model capturing the cascade of intracellular reactions that are essential for the initiation of the apoptosis. The model reactions are partitioned into two modules: a stochastic and a deterministic module (Fig 2).

The first stochastic module represents the process of stochastic assembly of DR-induced DISC/RIPoptosome multiprotein platform which facilitates initiation of ProCasp8 dimerization and self-activation by cleavage (Casp8*; activated Casp8 dimer in Fig 2E). We implemented this module with the direct Gillespie stochastic simulation algorithm [50,51] which accounts for molecular fluctuations and slow association and dissociation rates following each component of the platform individually. It assigns the reaction propensities in probabilistic terms. The binding propensities of ProCasp8 together with its binding partner protein, Fas-associated death domain protein (FADD), and competitor protein RIP1/RIP3 that comprise the core scaffold of RIPoptosome are calculated from the concentrations that we quantified experimentally in HeLa cell culture (Table D in S1 File). FADD protein is crucial for apoptotic initiation [35,52]. This protein consists of both a Death Effector Domain (DED) and Death Domain (DD) which are specific motifs for ProCasp8 [53] and RIP1 [16,54] self-oligomerization respectively. Through these domains, ProCasp8 and RIP1 are bridged via FADD (as shown in grey in Fig 2B). RIP3 protein can form homo-oligomers, but can also associate with RIP1 scaffolds through the RIP homotypic interaction motif (RHIM), forming amyloid structures [27,28] (Fig 2B). Intensive recruitment of RIP3 molecules to the amyloid triggers transphosphorylation of RIP3 by RIP1 with consequent transmission of phosphate groups to the MLKL pseudokinase. Phosphorylated MLKL executes necroptosis [25,30]. Therefore, in the absence of FADD and joint Casp8 activation platforms these structures spontaneously trigger necroptosis [35,55,56] (necrosome complex; purple in Fig 2B). Additionally, we quantified the concentrations of the cellular FLICE (FADD-like IL-1β-converting enzyme)-inhibitory protein (c-FLIP). As a DED-containing protein, cFLIP in its short (cFLIPs) and long (cFLIPl) form, can be recruited to the ProCasp8 platform abrogating or restricting activation of Casp8 [53,57,58] (cFLIP molecules; light and dark blue in Fig 2B). In addition to this suppression, Casp8 activation can be disrupted by binding its own processed DEDs which may remain in the cytosol (DED1-DED2; white in Fig 2B).

The second deterministic module mimics the activation of two effector caspases, Caspase 3 (Casp3) and Caspase 6 (Casp6) which is triggered by stochastically activated Casp8. Pro-forms of both caspases form stable dimers at physiological concentrations [59]. By cleavage, Casp8 activates Casp3 (Casp3*; activated dimer of Casp3 in Fig 2E) [60]. Casp3* activates Casp6 (Casp6*; activated dimer of Casp6 in Fig 2E) [61,62] and has autocatalytic function cleaving ProCasp3 [63,64]. Finally, Casp6* can cleave free ProCasp8 (Casp8*; cleaved monomer of Casp8 in Fig 2E) [64–67] however Casp8 becomes active only after a very slow dimerization (Casp8*) [19,21]. Previous models suggest that this effector caspase feedback upon weak DR stimulation probably can accelerate Casp8 activation which was initially started at the DISC or RIPoptosome platform [68]. However, the feedback can be inhibited by X-linked IAP (XIAP) which tightly binds Casp3 and, further, marks Casp3 with ubiquitin that leads to its proteasomal degradation [69,70]. The overall dynamics of Casp8 activation can be tracked quantitatively with a Casp8-specific FRET cleavage probe (FRET, Fig 2E). The fixed threshold rate of this FRET probe cleavage accurately determines the moment of MOMP in HeLa cells [2]. Based on the mass action and conservation laws, the time evolution of the variables that comprise this module were modelled by a deterministic system of ordinary differential equations (ODE) (details in S1 File).

All protein concentrations and parameters used in the model are provided in Tables D and E of Materials and Methods file (S1 File).

### Stochastic initiation of Casp8 activation through DISC/RIPoptosome assembly

**Fig 3:**
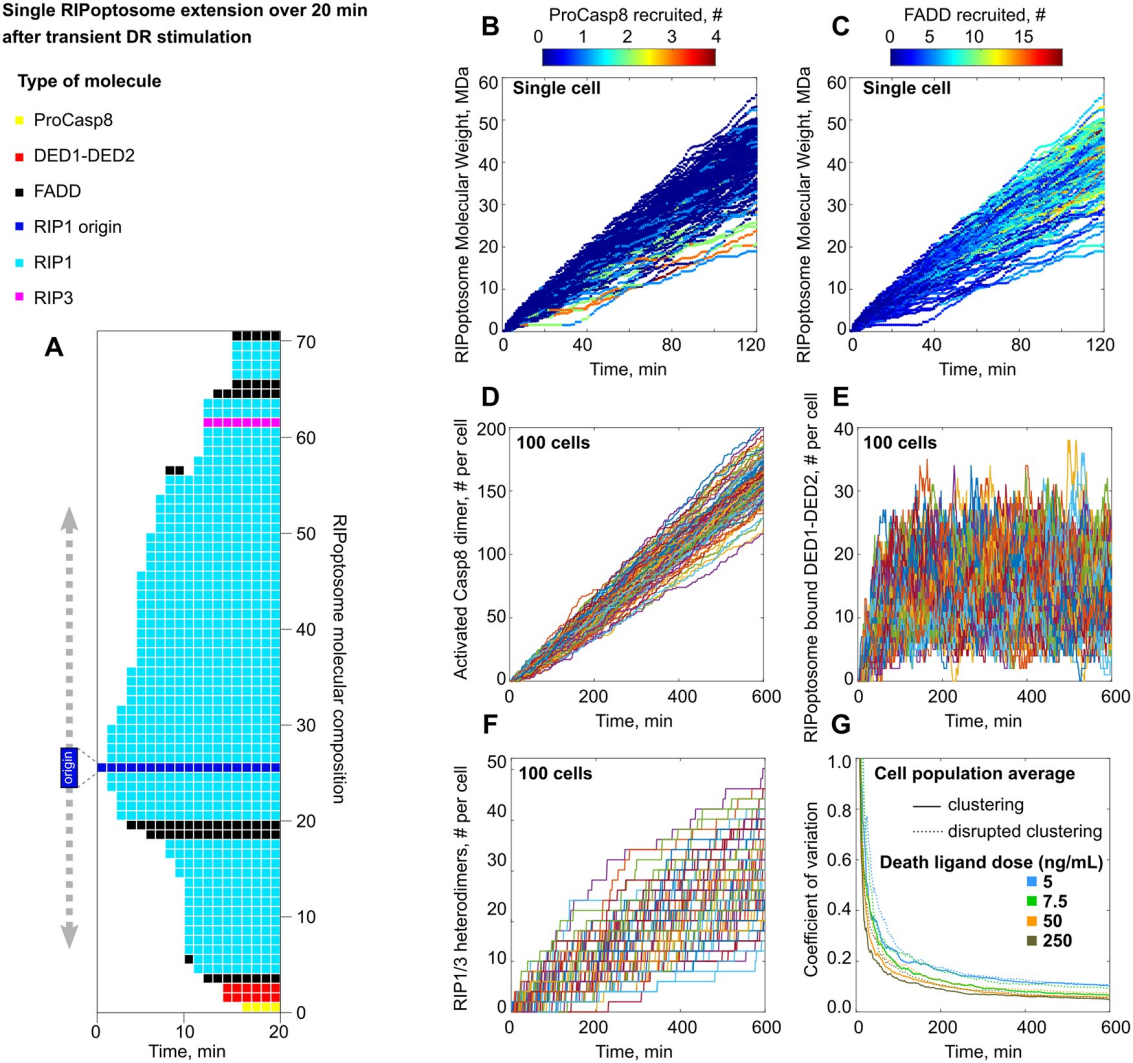
Simulated by model stochastic molecular assembly of RIPoptosome and Casp8, RIP1/RIP3 activation in a single cell. (A) Model generated individual RIPoptosome growth over first 20 min after DR stimulation with 5ng/mL of DL assuming receptor clustering scenario. (B,C) RIP1 filaments formation triggered immediately after DR stimulation which results in RIPoptosomes of size 2 MDa and higher within first 5 min. (B) ProCasp8 recruitment is mostly abundant in the filaments of the lower molecular weight. (C) FADD recruitment is proportional to the filament molecular weight and persistently increases with the filament progression. (D) Single cell stochastic traces of RIPoptosome driven Casp8 activation, (E) accumulation of DEDs (DED1-DED2) and (F) RIP1-RIP3 heterodimers induced by treatment with 5 ng/mL of the DL. 100 traces shown generated assuming DR clustering scenario. (G) Time dependant variability in Casp8 activation over population of 100 cells stimulated with DL doses of 5, 7.5, 50 and 250 ng/mL for regular (solid line) and disrupted (dashed line) receptor clustering order.

The estimated weight of the RIPoptosome after short DR-targeted stimulation may exceed 2MDa [10,24,29]. To reproduce the RIPoptosome growth and composition we first employed the stochastic modelling module simulating the assembly of the individual RIPoptosomes at the single cell level. RIP1 on its own forms unlimited filaments *in vitro* [28], however, in the cell the long-term RIPoptosomal filament growth is limited by the cell volume and the stiffness of the cellular components. We followed unlimited filament growths without implementation of these physical limits, focusing on the initial dynamics of RIPoptosome progression. Fig 3 and S2 Fig illustrates the simulated molecular composition of RIPoptosome in the single HeLa cell treated with a dose of 5 ng/mL of the DL (rhTRAIL).

The composition and the time evolution of individual RIPoptosomes within single cell differed from one to another. Consequently, the size and, therefore, molecular weight of those RIPoptosomes varies as well. As an example, we display the composition change in a few randomly chosen RIPoptosomes over the first 20 min with 1 min step interval (Fig 3A, S2 Fig). Next, we calculated the progression of the molecular weight of a complete cellular pool of RIPoptosomes as simulated by the model. Interestingly, we found a high degree of variation between the RIPoptosomes formed within the same cell (Fig 3B). Our simulations confirmed that in HeLa cells, the most populated protein within each RIPoptosome is RIP1 through its highly stable association mechanism. This is explained by the RHIM domain binding property that shares homology with β-amyloids assembly domains. Simulation of the model revealed that the RIP1 filaments formation is triggered immediately after DR stimulation (Fig 3A). The model also predicts that it would be possible to observe RIPoptosomes of size 2 MDa only 5 minutes after DR stimulation (Fig 3B).

FADD recruitment to the fraction of the high molecular weight complexes is persistently increasing with post treatment time [29]. Our simulations show as well that the abundance of FADD within a single RIPoptosome increases linearly with time progression (Fig 3C) in conjunction with the filament growth. As a result, the abundance of FADD on average will not exceed the amount of 10 molecules per origin within the first two hours. Moreover, this abundance is independent of DL dose. Thus, a low dose of 5 ng/mL of the DL and a high dose of 50 ng/mL will result in similar FADD abundance (S4A and S4B Fig).

On the contrary, ProCasp8 recruitment in the single cell is most abundant in the RIPoptosome of the lower molecular weight (Fig 3B). The binding of the ProCasp8 or its DEDs domain to the end of the filament blocks the RIP1 recruitment and therefore also blocks intensive filament growth by competition. The population average over 600 cells shows that ProCasp8 abundance per RIPoptosome (origin) saturates after 2 hours of stimulation (S4C and S4D Fig). This relative abundance does not vary significantly for doses of 5 or 50 ng/mL of the DL and is unaffected by the clustering or non-clustering assumption in the model. These rapid saturation dynamics of ProCasp8 compared to linear FADD translocation has been observed earlier in experiments where no co-binding of FADD and Casp8 has been observed after 1 hour of stimulation but has become apparent at the second hour [29].

### Single cell stochastic RIPoptosome assembly is the source of variation in Casp8 and RIP1/3 protein activation

Molecular fluctuations in the RIPoptosome composition within single cells cause the fluctuations in the active Casp8 abundance (Fig 3D). Stochastic single cell Casp8 activation traces for 5 ng/mL dose simulation with the corresponding per cell accumulation of Casp8 Pro domain (DED1-DED2) are shown in Fig 3D and 3E. Interestingly, limited expression of RIP3 [28] protein in HeLa cell gives rise to very low and therefore heterogeneous distribution of RIP1-RIP3 heterodimers among the cells (Fig 3F) making the spontaneous event of the necroptosis less probable to overtake the apoptotic course of the cell death.

Averaged over the population the Casp8 activation time course demonstrated high dependence on the dose of the DL as well as the clustering capacity (S3 Fig). Thus, even low doses of the DL with enhanced clustering property can activate Casp8. This result confirms the established success in the application of combinational therapeutics where the DL has been combined with the ligand specific cross-linking antibodies that enhance receptor clustering [49].

As expected, the overall variability in the Casp8 activation is a function of the treatment dose (Fig 3G). Despite the coefficient of variation being within the limits of low-variance (less than 1), the early Casp8 initiation dynamics can bring significant stochasticity into triggering the downstream death pathway. Interestingly, the enhanced receptor clustering did not reduce the variability in the individual HeLa cell Casp8 activation dynamics significantly. We observed only a minor decrease in the coefficient of variation over all tested conditions (Fig 3G).

### Deterministic modelling of effector caspases feedback into Casp8 activation

Next we studied the downstream caspase cleavage cascade, the second deterministic modelling module (Fig 2E), which feedbacks to the DISC/RIPoptosome based Casp8 production and is potentially capable of boosting cell apoptotic capacity especially following treatment of low doses of DL [68]. As an input we used the population average of the stochastic traces (Fig 2D, S3 Fig) we simulated for the first module of the DISC/RIPoptosome based network initiation assuming DR clustering (Fig 2B). Thus, we merged two modules into one complete deterministic system (Fig 2C) which enabled us to adjust undetermined parameters and estimate parameter sensitivity, hence avoiding computationally expensive parameter scans of the full stochastic formalism (see Materials and methods, S1 File).

**Fig 4:**
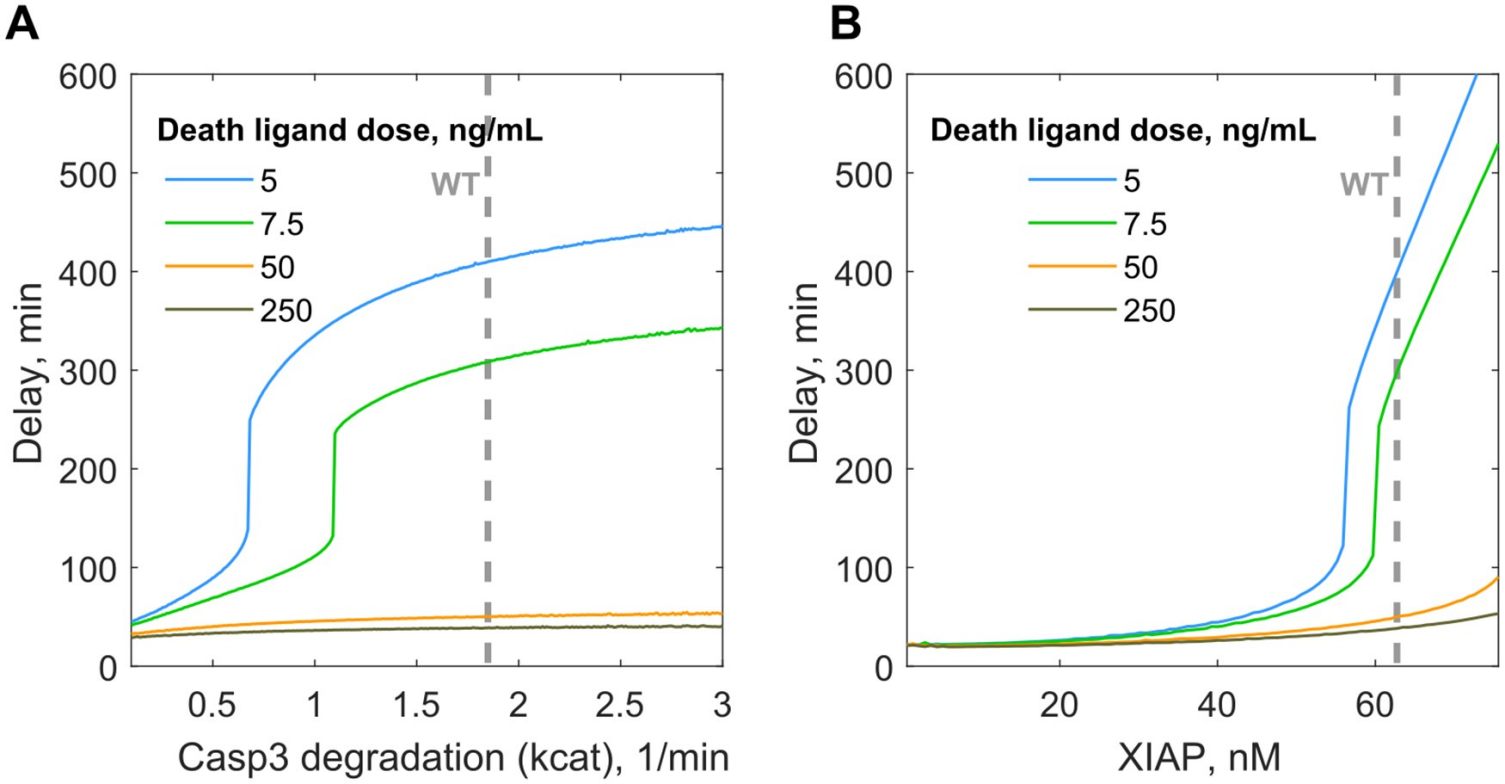
Cell death delay sensitivity to the change in ProCasp6 concentration and Casp3 degradation kinetics in the clustering scenario. (A) Dramatic increase in the cell death delay upon the increased Casp3 degradation rate constant (kcat) by XIAP mediated ubiquitination which is a function of proteasomal protein degradation capacity as well as Casp3 self-cleavage for the cells stimulated with lower DL doses (5, 7.5 ng/mL). (B) High sensitivity to the minor decrease in XIAP concentration from its mean estimated concentration in HeLa cell 63 nM will lead to dramatic decrease in cell death delay for the cells stimulated with lower DL doses.

The first undetermined parameter is the rate constant of Casp3 ubiquitin dependent degradation (*kcat*). Ubiquitination of active Casp3, which is set by XIAP, will attract proteasomal complex leading to Casp3 degradation. However, application of proteasome inhibitors does not stabilise the pool of active Casp3 and consequently does not result in reduced Casp3 proteasomal degradation. Instead, Casp3 catalytic activity is absolutely required for its own proteasomal degradation [71,72]. Therefore, dynamics of Casp3 degradation triggered by XIAP will not match the general degradation dynamics triggered by ubiquitin ligases for other types of proteins and this specific rate constant needed to be identified. We estimated that *kcat* needs to be significantly higher (1.75 min^-1^) from the general (basal) ubiquitin-dependent degradation rate (0.04 min^-1^) [73] (Table E in S1 File). Again, low doses of the DL bring into play a switch-like sensitive response to the change in *kcat* value (Fig 4A). In this case the cell death delay can be initiated in a spontaneous fashion if the Casp3 degradation mechanism is perturbed.

Furthermore, the similar steep ultra-sensitive response can be also initiated by the mild fluctuations in the XIAP concentration. We found that slight deviations from the mean XIAP level, 63 nM, quantified earlier for HeLa [1] could speed up the cell death by more than 3-fold in the case of low DL doses (Fig 4B). This decrease could be very sudden through this switch-like type of response. Indeed, XIAP specific inhibitors such as Embelin, Mithramycin A are able to overcome the DL resistance in different cancer types [74,75].

### Semi-stochastic hybrid modelling of the complete initiation network

Finally, with the fully identified parameter set we formulated the new semi-stochastic hybrid model of apoptotic pathway initiation in a single cell with the fixed partitioning of the whole network into discrete (Fig 2B) and continuous reactions (Fig 2E). The slow discrete reactions are the DISC/RIPoptosome assembly. The fast continuous reactions capture the caspase cleavage cascade.

**Fig 5:**
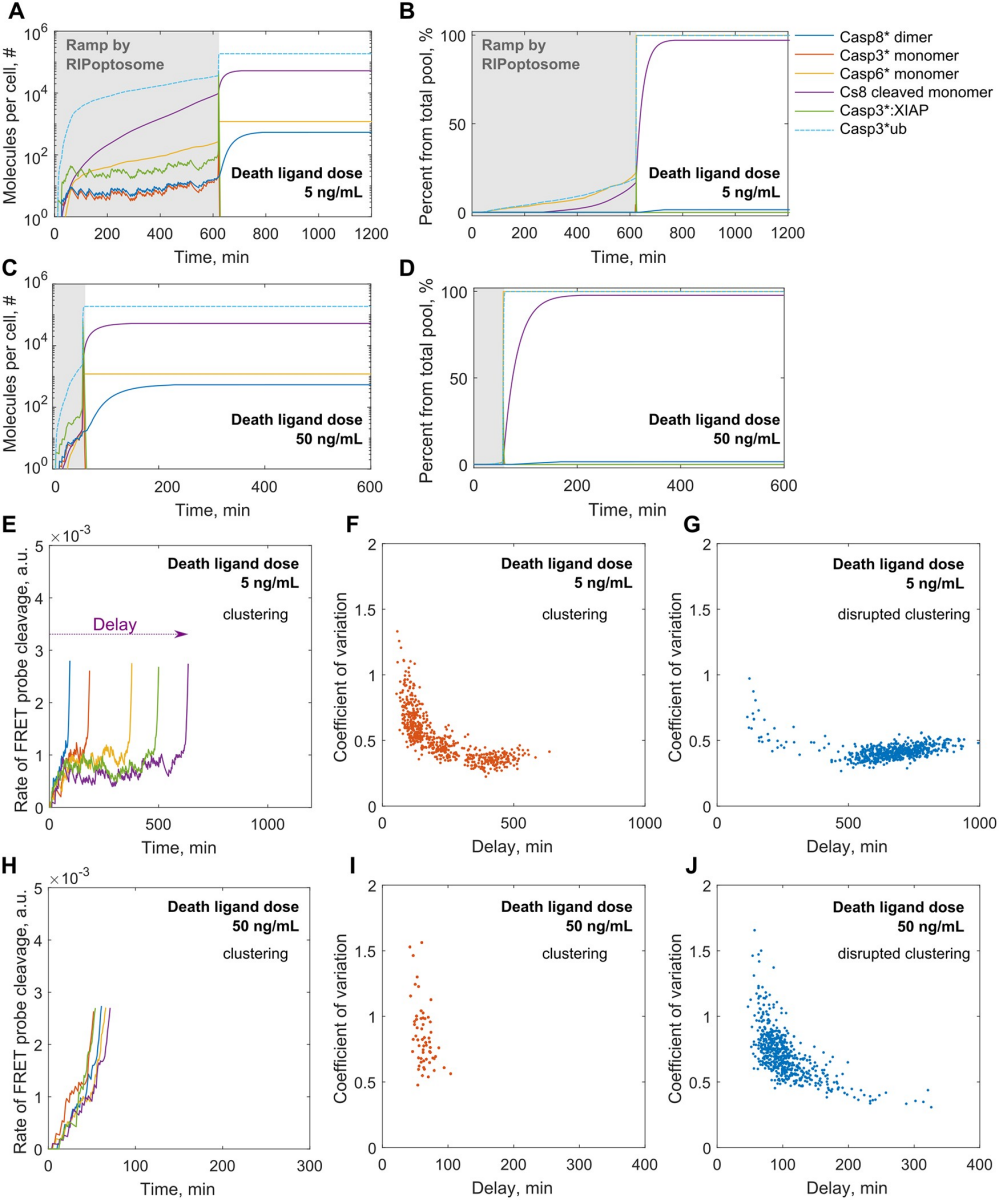
Simulated by semi-stochastic model single cell network activation. (A-D) Plots represent single cell responses to the low, 5 ng/mL, and high, 50 ng/mL, DL treatment doses. Responses begin with the slow ramp regime (in grey) characterised by high stochastic noise explained by random assembly of RIPoptosome. Once a sufficient rate of Casp8 activation that can overcome XIAP dependent Casp3 inactivation is reached, a rapid switch transition is triggered through the positive caspase feedback loop. The molecular number of active Casp8 dimers; active Casp3, Casp6 and Casp8 monomers; XIAP and ubiquitin targeted Casp3 molecules per HeLa cell are reflected (A, C) as well as theirs percentage from the total initial pool (B, D). (E-J) Plots represent cell death delay driven by intrinsic noise. Single cell stochastic ramp traces of FRET probe cleavage are shown for the five randomly chosen cells simulated with the low, 5 ng/mL, (E) and high, 50 ng/mL, (H) DL treatment doses upon receptor clustering. The trajectories were terminated at the point of death threshold rate (see S1 File). Scatter plots represent the relation between individual cell ramp noise and time of cell death delay within the population of 600 cells for the low, 5 ng/mL, (F-G) and high, 50 ng/mL, (I-J) DL doses with the normal (F, I) and disrupted (G, J) receptor clustering order.

The simulation results for a single cell response on the addition of low and high amounts of the DL are demonstrated in Fig 5. We observed a prolonged ramp effect for all variables of the network before the system switched to the rapid response. The ramp duration for the displayed example exceeded 10 hours after treatment with the low dose of the DL (Fig 5A). Whereas the high dose treatment stimulates the ramp for shorter times, around one hour for a shown example (Fig 5C). In a similar manner to the simulations with our entirely deterministic model, the delay for the switch in the single cell response is a function of DL dose (Fig 4).

However, for both high and low doses we also observed very high dynamic noise in the ramp (Fig 5A and 5C). This noise characterises the time course of dimeric Casp8 and active Casp3 accumulation. In experiments both proteins are very unstable and hardly detectable in the pre MOMP period of apoptotic initiation [19,71]. As we have shown earlier initial formation of new Casp8 dimer species can be limited by the vulnerability in molecular assembly of the DISC/RIPoptosome platform (Fig 3). Moreover, active Casp8 dimer is unstable due to high dissociation rate in the cytoplasm [19,20]. Indeed, Casp8 under physiological concentrations is found mainly in monomeric form [18,20,59] (Fig 5B and 5D). Therefore, this process prevents accumulation of the excess catalytically active pool of Casp8 for further downstream apoptotic signalling in the pre MOMP period.

Casp3, as the main Casp8-dependent effector caspase [60], follows the noise in the dynamic course of Casp8 dimer during the ramp. Besides, Casp3 is sacrificed in the pre MOMP period due to the excess amount of XIAP which effectively [1,76] blocks Casp3 activity by binding and subsequent ubiquitination which leads to Casp3 degradation.

To study how the ramp noise property in individual cells influences the cell death delay we have performed 600 independent simulations of the semi-stochastic model mimicking the overall cell culture response. These simulations were repeated for four different scenarios: low and high dose treatment scenarios with or without receptor clustering order. The coefficient of variation in Casp8 dependent FRET probe cleavage calculated over ramp period for each cell was considered as the measure of the noise strength. As earlier, the moment of the individual cell death was recorded once the rate of FRET probe cleavage exceeded the expected experimental threshold rate [2] (Fig 5E and 5H). For the individual cells treated with low dose the cell death delay varied from 1 to 10 hours if we integrated the receptor clustering order. Even higher variability was observed when the clustering was absent. In this case the cell death time could vary from 1 to 22 hours. Examples of FRET time traces for five individual cells are shown in Fig 5E. By visualising the relationship between the single cell death delay and dynamic ramp noise strength over a population, we found out that noise was an important determinant of the delay. For both clustering and non-clustering scenarios this relationship follows the same trend (Fig 5F and 5G). Moreover, this trend was independent of the treatment dose (Fig 5I and 5J). Furthermore, for all tested scenarios coefficient of variation higher than 0.5 strictly characterised early dying cells which commit apoptosis within the first 2 hours. Interestingly, receptor clustering enhanced ramp noise resulting in higher values of coefficient of variation (Fig 5F and 5I).

**Fig 6:**
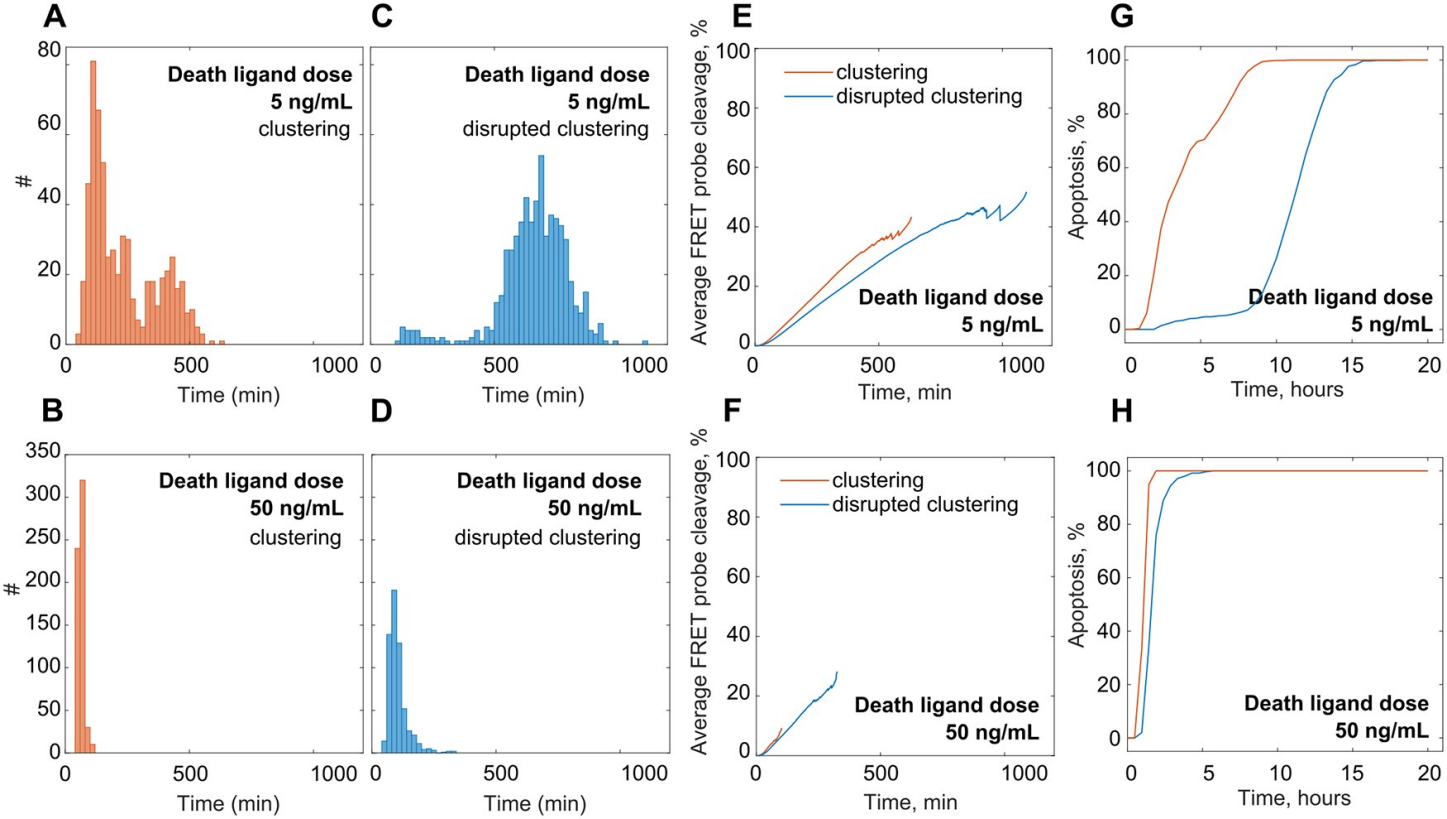
Low DL dose induced bimodal distribution of death delay within the cell culture. (A-D) Plots demonstrate the frequency distribution of death delays over the population of 600 cells simulated under low (A,C) and high (B,D) DL treatment doses under receptor clustering (A,B) and disrupted receptor clustering (C,D) assumption. (E-H) Casp8 activation characteristics calculated from the population average are not indicative for the apoptotic rate. (E) and (F) show population average dynamics of the Casp8-dependent FRET probe cleavage for the population of 600 cells simulated under low and high DL dose scenarios under receptor clustering (red line) and disrupted receptor (blue line) clustering assumption. (G) and (H) show the cumulative apoptosis event occurrence in the cell population.

Fractional cell killing was observed in DR-targeted treatments especially when applied in low amounts [2]. As we have shown, the high dispersion of the death delays was the main reason for fractional cell killing. What we found more interesting is that dispersion of the delays could exhibit strong bimodality clearly distinguishing between the fraction of early and late dying cells. Clear bimodality was predicted by our model particularly for the low ligand dose upon receptor clustering order (Figs 6 and 5A). Taking this fact together with the ramp noise analysis (Fig 5F) we can conclude that the high noise in the ramp sensitises cells for early death which will take place within the first five hours at the latest. This fluctuation-enhanced sensitivity has been called ‘stochastic focusing’ and allows quicker system relaxation to the stationary state when the noise is high. The bimodality breaks when receptor clustering is interrupted (Fig 6C, see S1 File) and most of the cells would die only after 10 hours. On the population average cell dynamics receptor clustering provides slightly quicker Casp8 activation for the low dosage of the DL (Fig 6E). This may enable better coupling of this stochastic process with the continuous positive caspase feedback loop. Thus, stochastic focusing coupled with the positive feedback facilitates a more robust bimodal response without the need of multi-stability encoded in the system itself. Finally, the overall cell survival can be dramatically reduced by enhancing the receptor clustering mechanisms (Fig 6G).

## Discussion

The roles of multiprotein signalling platforms assembled upon DR stimulation have been broadly discussed in the context of the programmed cell death initiation [11,17,29,31] as well as proliferation and proinflammatory signalling [5,77] over the last decades. The effect of DR and apoptotic inhibitors targeting on the structure and function of these platforms were investigated in different experimental models. However, the mechanism through which these platforms give rise to distinct functions is still poorly understood. Particularly, the mechanism through which the heterogeneous apoptotic response to the DR targeted therapeutics is initiated and how it can explain fractional cell death remains unclear. Our study shows that the noise exclusively caused by the stochastic molecular assembly of the DISC/RIPoptosome platform is able to explain fractional cell killing at low receptor level engagement. Furthermore, this noise in conjunction with receptor clustering facilitates a more rapid apoptotic response.

Most of the variability in cell death delay raised upon DR stimulation originates from the pre-MOMP phase. Individually, none of the proteins involved in the apoptosis activation prior to MOMP can explain variation in cell death delays. Casp8 activation rate and consequently the rate of Casp8-dependent BID cleavage are the only determinants of the process [2,4,78]. Casp8 activation is entirely dependent on the assembly of the multiprotein signalling platform such as RIPoptosome. Though there have been a few models developed none have explicitly accounted for the stochastic nature of the signalling platform assembly [79]. Hence in this study, we developed a novel mathematical model of the stochastic assembly of the RIPoptosome in the single cell together with downstream effector-caspases cascade. Two of these processes are paired together in the pre-MOMP phase of apoptotic pathway initiation. By incorporating the absolute protein concentrations that we have measured in HeLa cells experimentally, and using kinetic parameters derived from the literature we have simulated the Casp8 activation dynamics in the single cell for various conditions: different DL doses, full and disrupted DR clustering propensity. Our modelling simulations have shown that the random and competitive multiprotein assembly of RIPoptosome allows prolonged and slow activation of Casp8 in a ramp-like fashion which is prone to high stochastic fluctuations. Such fluctuations in conjunction with downstream positive feedback loop of effector caspases after certain delay can lead to the spontaneous acceleration of Casp8 accumulation. Because of these fluctuations each cell behaves differently. We have found that the time the single cell will commit to apoptosis depends on the amount of intrinsic noise level in the initial ramp Casp8 activation. The higher ramp noise favours quicker cell death. By that we provide the evidence that the random assembly of RIPoptosome on its own, without any contribution of extrinsic noise in protein expression may explain the heterogeneous cell death response.

Our modelling predictions confirm that the receptor clustering process is critical in the extrinsic apoptotic response initiation [80]. Furthermore, a lower DL treatment dose will benefit the most from the enhanced clustering capacity over all. However, the significant fraction of the cell population will remain in the delayed apoptotic state. This new finding is clearly reflected in the bimodality of the distribution of death delays initiated by low DL dose where we demonstrated the clear split of the cell population into early and late responders (Fig 6A).

Despite the high affinity of XIAP to Casp3, their concentration balance in HeLa cell does not ensure robust Casp3 inhibition prior to MOMP [76]. Additionally, XIAP stimulated Casp3 ubiquitination that leads to Casp3 degradation is critical to keeping the downstream executioner caspases cascade shut till the MOMP is set. We have shown that for the fixed XIAP level in HeLa, Casp3 will play an important role in determination of cell death delay. Thus, suppression of the Casp3 ubiquitination/degradation rate at some point can trigger an ultra-sensitive switch from late to early cell death (Fig 4A). This response is characteristic for the low doses of DL and has been suggested in previous modelling studies [68]. However, experimentally Casp3 proteasomal degradation is hard to inhibit unless the catalytic activity of Casp3 is suppressed [71,72]. Instead, XIAP inhibition can initiate the same effect (Fig 4B) and as we showed very minor suppression is needed to return rapid cell death response initiated by subminimal DL doses. We believe that this ultra-sensitivity serves the best explanation for established success in the application of XIAP specific inhibitors for DL dependant cell death amplification [74,75,81,82]. Strikingly, we found that at the low DL doses an increase in XIAP level exclusively would cause a tremendous linear increase in the time of cell death delay. Indeed, exceptionally only XIAP overexpression, not cIAP1/2 or Smac up and down regulation respectively, is the apoptosis resistance mechanism which can be developed in cancer cells in response to the chemotherapeutics [83].

The content and dynamics of the RIPoptosome assembly predicted by model conform the general knowledge that RIP1 is the most abundant protein among all that are comprising the core RIPoptosome scaffold [10,23,24,54,84–86]. The engraftment of ProCasp8 molecules into RIP1 oligomer can happen when the RIP1 filament growth is interrupted by binding of single FADD molecule that occasionally can lead to the sequential binding of ProCasp8. Our simulations have showed that this event is very rare for a given level of RIPoptosome proteins in HeLa cell and we do not see strong oligomerization of ProCasp8 or its DEDs in HeLa cell. Despite, overexpressed truncated form of ProCasp8 which includes only DED1-DED2 domain is prone to form filamentous structure by oligomerisation [12,53,87,88], the full length protein do not oligomerize [87–89]. Overall, the quantitative balance between the components may dictate the structure of the RIPoptosome that vary between different cell types [12,84,85]. Therefore, we can conclude that the RIPoptosome formation in HeLa is a competitive process of RIP1, FADD and ProCasp8 assembly and the structure and function of this assembly varies due to the noisy nature of the core protein binding and dissociation events.

**Fig 7:**
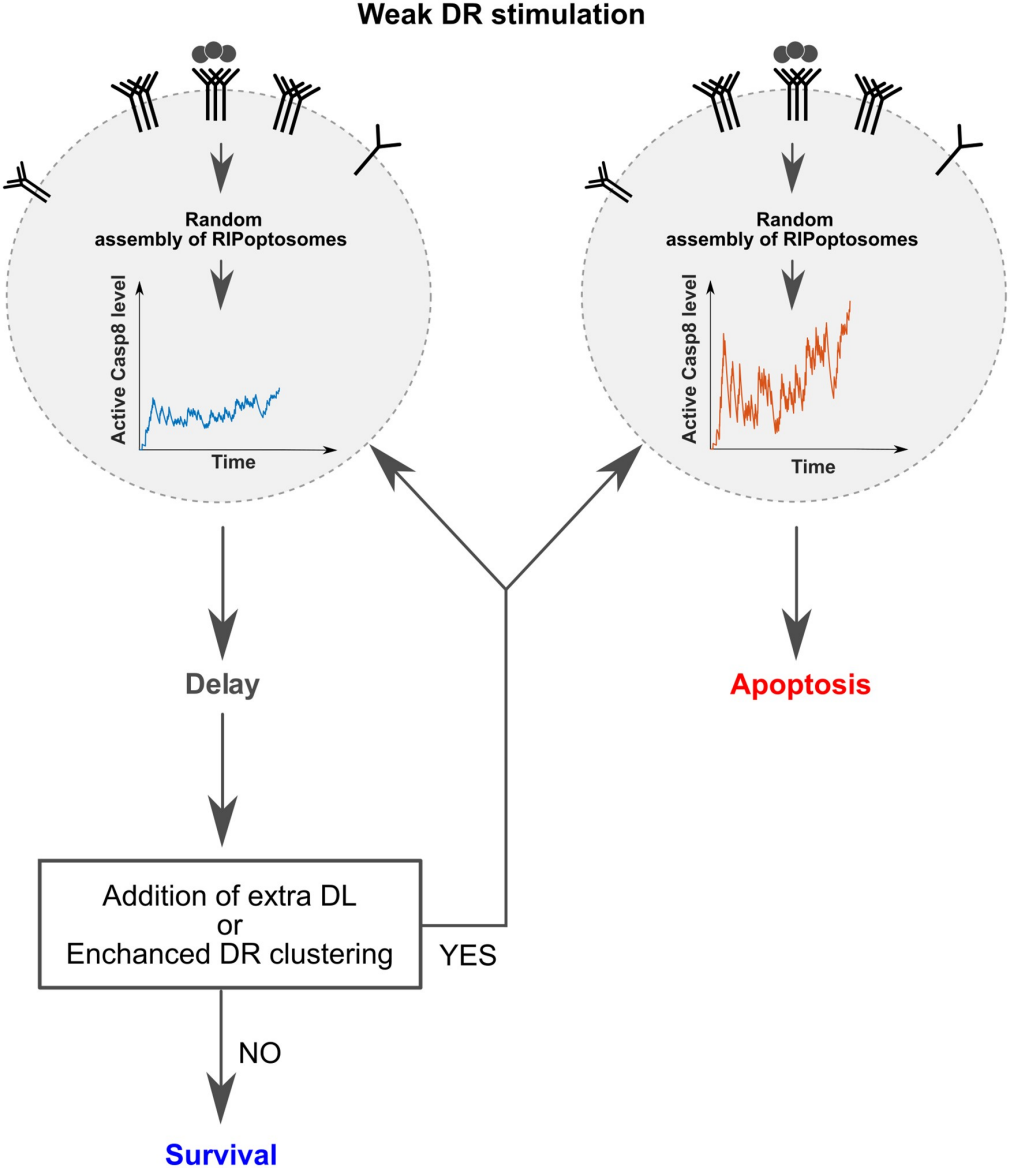
Representation of how stochastic assembly of the RIPoptosome yields kinetic proofreading of weak DR stimulation for individual cell. Figure represents two cell decisions. One of which, apoptosis, is taken because of the RIPoptosomes higher capacity to reconstitute Casp8 activation quicker and the signature of that is the high noise (red curve). The other decision is to wait or delay apoptotic cell response till more sophisticated DR signalling because the current RIPoptosomes are not efficient enough to maintain robust Casp8 activation rate which is confirmed by low noise (blue curve).

In this context, the slow and probabilistic nature of Casp8 activation explained in our current study by the random RIPoptosome assembly serves as the basis for caution mechanism of kinetic proofreading. This mechanism needs to be in place to verify weak or temporal apoptotic stimuli. The cells which succeeded to assemble the pool of RIPoptosomes that can sustain efficient Casp8 activation will proceed further down the apoptotic pathway triggering MOMP. The high noise in the ramp of Casp8 activation, in this case, will signify the high RIPoptosome efficiency showing that each moment the Casp8 activity is sacrificed the next moment it can be reconstituted or even amplified (Fig 7).

The vulnerability of the apoptotic pathway and its susceptibility to adaptation are currently the key limitation of therapeutics designed to kill cancer cells through the DR targeting therapeutics. In this paper, we have uncovered the original mechanism that explains inefficient cell death stimulation through stochastic activation of apoptosis initiating caspase signalling, leading to heterogeneous responses. We believe that detailed understanding of basic principles of early events of cell death initiation may also stimulate more rationalised approaches in the development of combinational treatments against cancer.

## Methods

### DR amount, proteins concentrations and kinetic parameters determination in HeLa

We quantified TNFR1 in HeLa cells by QuantiBRITE phycoerythrin beads based assay. The amount of DR4/5 was calculated from TNFR1 level by comparative MS data analysis (Table A in S1 File). Receptor clustering conformation was calculated from experimentally derived cluster size probability distributions (S1 Fig). Initial protein concentrations were taken from the literature (Table D in S1 File). Except FADD and RIP1, which we quantified with recombinant protein comparative Western Blot and ProCasp6 concertation that we adjusted using the complete deterministic model (S1 File). Most binding kinetics and catalytic enzymes activity parameters were retrieved from the literature (Table E in S1 File). Hence FRET probe cleavage rate and Casp3 degradation rate were adjusted in the simulations.

### Mathematical model

Modelling formalism of Gillespie stochastic simulation algorithm (SSA) and ODE integration as well as semi-stochastic hybrid model was implemented in the MATLAB 2017b environment (see also S1 File).

## Supporting information

S1 Fig Trimeric DR clustering. Experimental frequency distribution of the TNFR1 cluster size on the cellular membrane of unstimulated (black line) and TNFα stimulated HeLa cells derived by Super-resolution PALM microscopy (Fricke *et al*., 2014). Distribution for ligand free (in blue) and ligand bound receptors (in pink) in stimulated cells are followed separately. Distribution of bound receptors was approximated by splines (in red). Distribution for the trimeric Vesicular Stomatitis Virus G protein (VSVG) (in yellow) was used for the peaks calibration. Numbers above the peaks represent amount of monomeric receptor per corresponding cluster and red dots below represent the trimeric receptor complexes. The table demonstrates the conversion of the frequencies into the percentage of clusters from the total DR pool.
(TIF)

S2 Fig Simulated by model stochastic molecular assembly of RIPoptosome and Casp8, RIP1/RIP3 activation in a single cell. Model generated individual RIPoptosome growth over first 20 min after DR stimulation with 5ng/mL of DL assuming receptor clustering scenario. Plots show eight individual RIPoptosomes randomly chosen from different randomly selected cells.
(TIF)

S3 Fig RIPoptosome based Casp8 activation on the cell population average level as computed from ensemble simulations of stochastic model. Population average Casp8 activation per cell simulated with model under receptor clustering and disrupted receptor clustering assumption upon stimulation with 5, 7.5, 50 and 250 ng/mL of the DL. Single cell trajectories have been averaged over 100 cells in each represented condition.
(TIF)

S4 Fig FADD and ProCasp8 abundance within RIPoptosome. Average FADD abundance per origin simulated for culture of 600 HeLa cells with clustering scenario (A) and without clustering (B) for low (5 ng/mL) and high (50 ng/mL) concentrations of the DL. (C) and (D) represent corresponding quantities for average abundance of ProCasp8 together with DED1-DED2.s.
(TIF)

S5 Fig Comparison of semi-stochastic hybrid model with full SSA. Result of semi-stochastic model (A, B) and full stochastic model (C, D) simulation for 20 HeLa cells with clustering scenario for 50 ng/mL concentration of the DL.
(TIF)

S1 File Materials and methods. (PDF)

S1 Appendix A Matlab code for semi-stochastic model simulation. (M)
