# Supplementary figures and images for: Heterogeneous responses to low level death receptor activation are explained by random molecular assembly of the Caspase-8 activation platform
Source: PLoS Comput Biol. 2019 Sep 25;15(9):e1007374. doi: 10.1371/journal.pcbi.1007374 (PMC6779275; doi:10.1371/journal.pcbi.1007374)

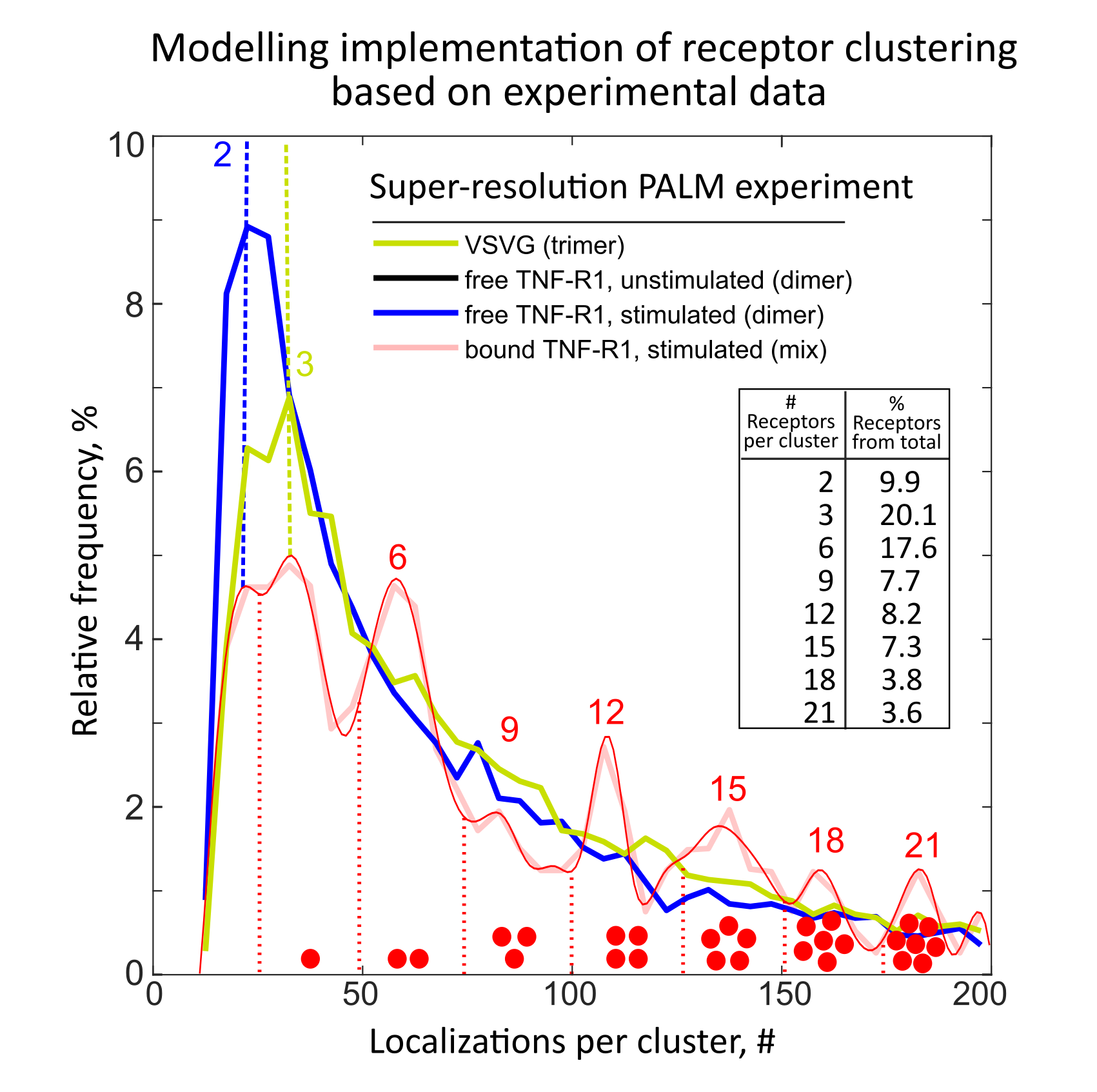

Supplement: S1 Fig — Experimental frequency distribution of the TNFR1 cluster size on the cellular membrane of unstimulated (black line) and TNFα stimulated HeLa cells derived by Super-resolution PALM microscopy (Fricke et al., 2014). Distribution for ligand free (in blue) and ligand bound receptors (in pink) in stimulated cells are followed separately. Distribution of bound receptors was approximated by splines (in red). Distribution for the trimeric Vesicular Stomatitis Virus G protein (VSVG) (in yellow) was used for the peaks calibration. Numbers above the peaks represent amount of monomeric receptor per corresponding cluster and red dots below represent the trimeric receptor complexes. The table demonstrates the conversion of the frequencies into the percentage of clusters from the total DR pool. (TIF) [file pcbi.1007374.s001.tif]

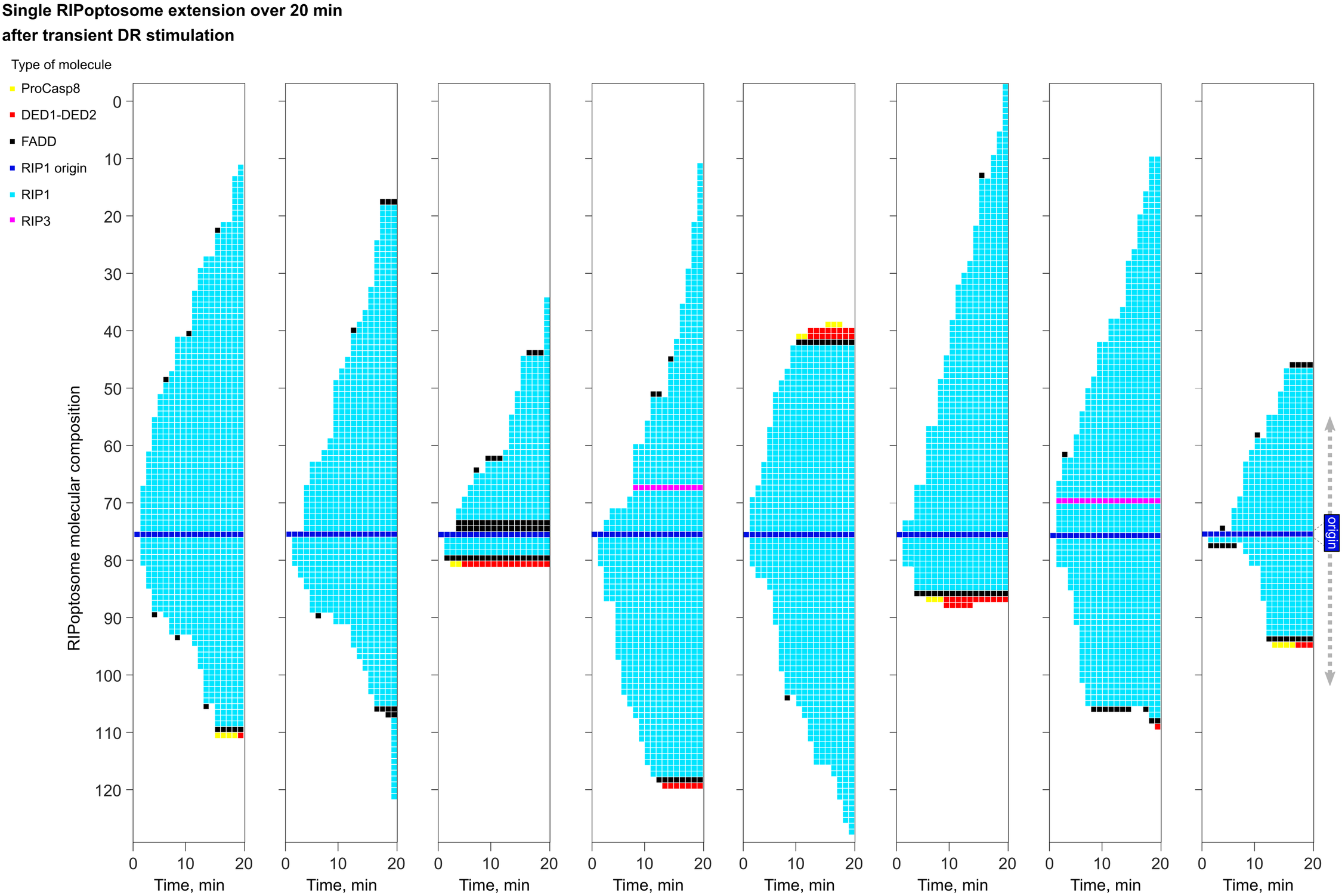

Supplement: S2 Fig — Model generated individual RIPoptosome growth over first 20 min after DR stimulation with 5ng/mL of DL assuming receptor clustering scenario. Plots show eight individual RIPoptosomes randomly chosen from different randomly selected cells. (TIF) [file pcbi.1007374.s002.tif]

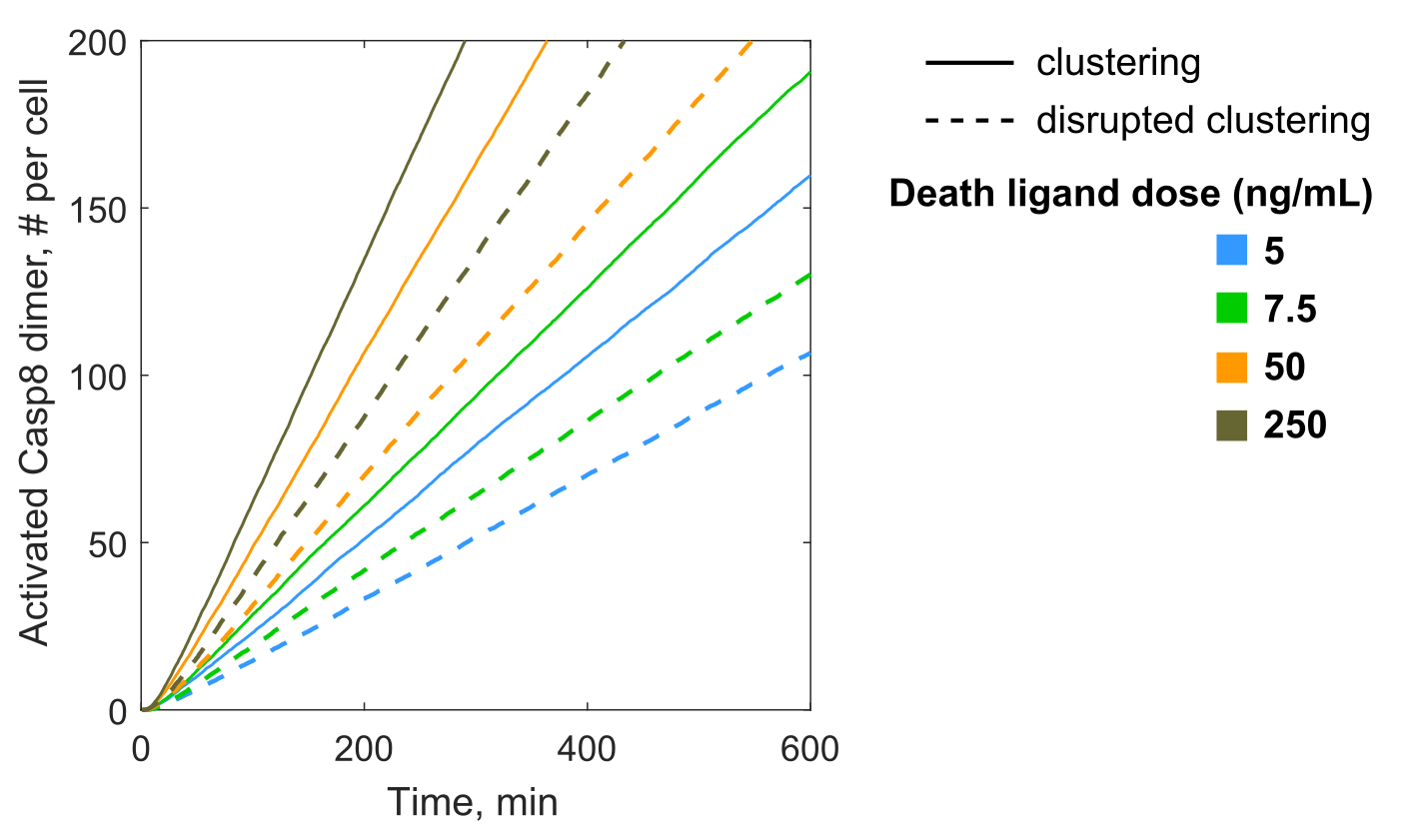

Supplement: S3 Fig — Population average Casp8 activation per cell simulated with model under receptor clustering and disrupted receptor clustering assumption upon stimulation with 5, 7.5, 50 and 250 ng/mL of the DL. Single cell trajectories have been averaged over 100 cells in each represented condition. (TIF) [file pcbi.1007374.s003.tif]

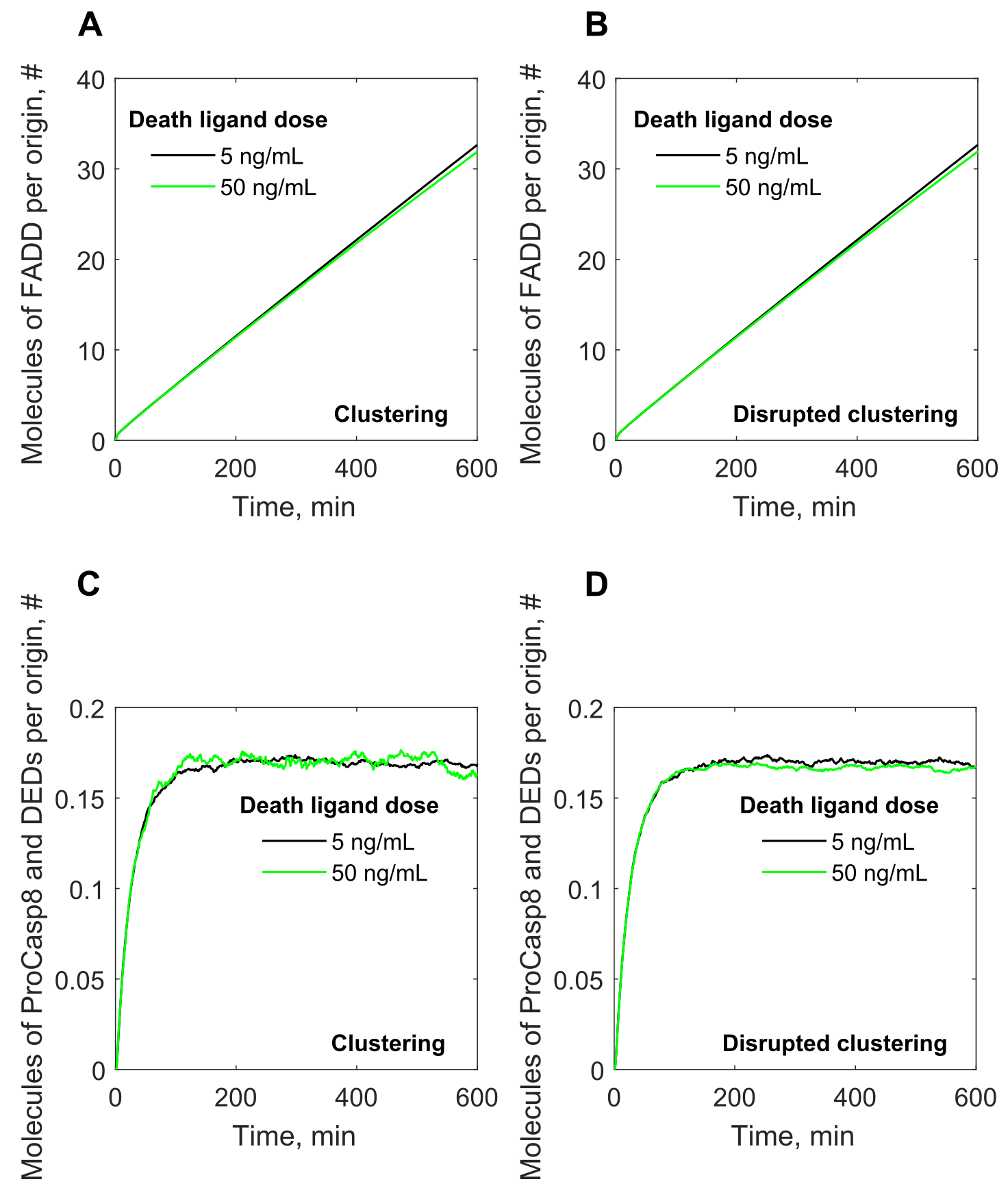

Supplement: S4 Fig — Average FADD abundance per origin simulated for culture of 600 HeLa cells with clustering scenario (A) and without clustering (B) for low (5 ng/mL) and high (50 ng/mL) concentrations of the DL. (C) and (D) represent corresponding quantities for average abundance of ProCasp8 together with DED1-DED2.s. (TIF) [file pcbi.1007374.s004.tif]

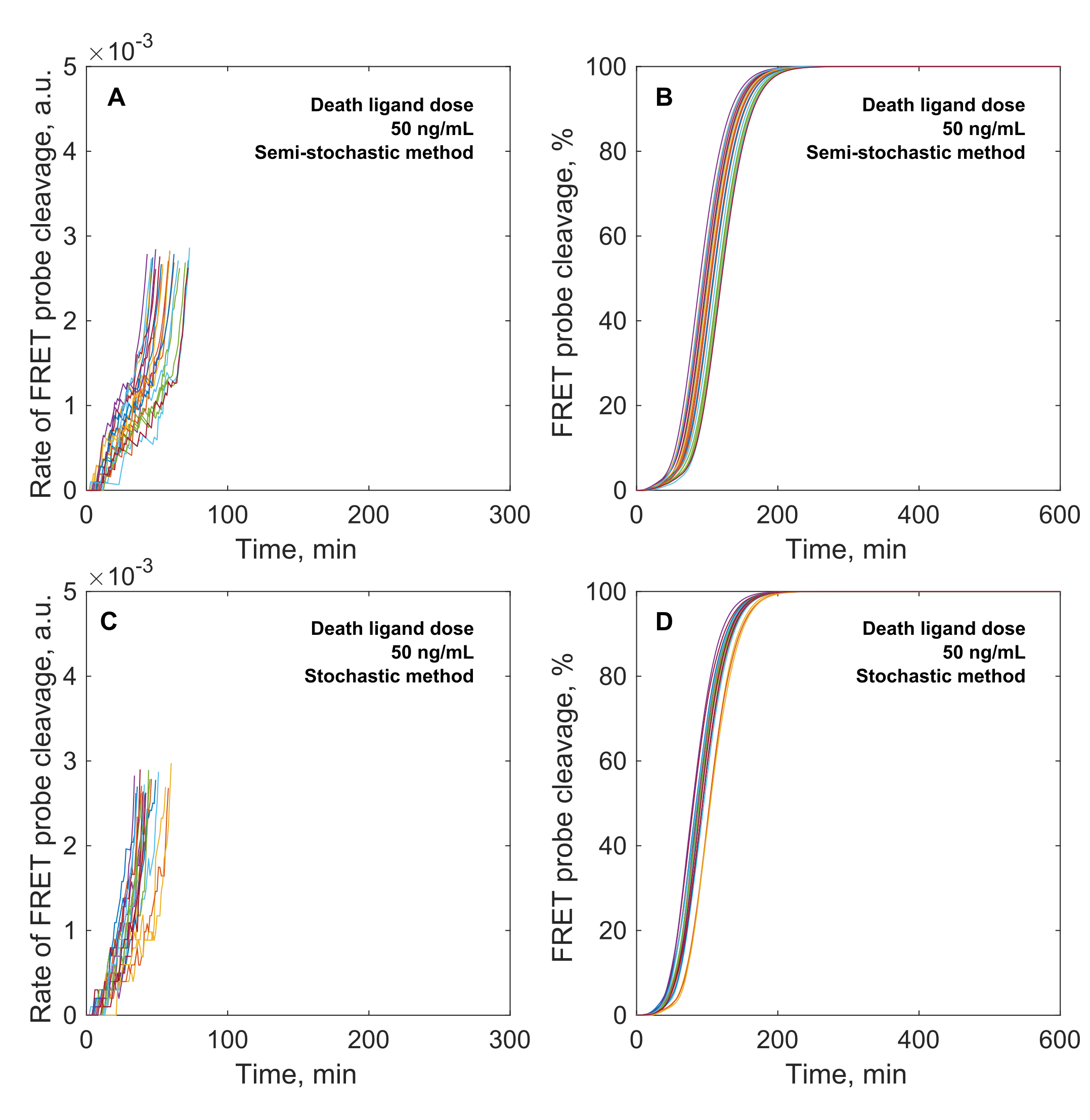

Supplement: S5 Fig — Result of semi-stochastic model (A, B) and full stochastic model (C, D) simulation for 20 HeLa cells with clustering scenario for 50 ng/mL concentration of the DL. (TIF) [file pcbi.1007374.s005.tif]
