# Supplementary material for: Heterogeneous responses to low level death receptor activation are explained by random molecular assembly of the Caspase-8 activation platform
Source: PLoS Comput Biol. 2019 Sep 25;15(9):e1007374. doi: 10.1371/journal.pcbi.1007374 (PMC6779275; doi:10.1371/journal.pcbi.1007374)
Supplement: S1 File — (PDF) [file pcbi.1007374.s006.pdf]

Supporting File 1: Materials and methods

QuantiBRITE phycoerythrin beads based assay for TNFR quantification

HeLa cells were cultured in DMEM supplemented with 10% FBS and Penicillin/Streptomycin. The cells were detached with TrypLE express (Thermo Fisher Scientific) and incubated in a 50ml tube (Sarstedt) for 30 min at 37 °C and 5 % CO2. The PE-labelled monoclonal anti-CD120a and anti-CD120b antibody (Biolegend; Lot: B230531 and B226634, respectively) was diluted 1:10 in PBS with 2% FBS. The cells were counted, 100,000 cells were resuspended in the CD120a or CD120b staining solution and incubated for 20 min on ice in the dark. Afterwards, the cells were washed with PBS + 2% FBS and resuspended in 200 µl PBS + 2% FBS. To quantify the amount of bound antibodies per cell the QuantiBRITE PE Quantitation Beads (BD Biosciences, UK) were used according to manufacturer's instructions. A BD LSR II (BD Biosciences) was used for data acquisition and analysis was done using the FACS Diva software (Version 5, BD Biosciences).

Mass spectrometry based quantification of death receptor amount

Mass spectrometry (MS) derived Intensity-Based Absolute Quantification score (iBAQ) values of protein content hold a strong power law correlation with the absolute protein amount over at least four orders of magnitude [1]. Taking into account that protein expression of death receptors, TNFR1 and DR4/5, is correlated with the receptor abundance on the cell surface [2], we can derive the absolute quantities for DR4/5 by comparing their iBAQ scores with the iBAQ score of TNFR1 [3] and its absolute amount on the cellular membrane that we measured. The correlation between protein content and iBAQ protein scores is most accurately described by a log-log regression model:

$$\log_{10}(amount\ of\ X) = \alpha + \beta * \log_{10}(iBAQ\ of\ X)$$

However, in the absence of reference proteins, we were unable to estimate the α and β parameters therefore we have assumed the direct proportionality of protein absolute abundance to the iBAQ score as one of the common practices in MS data analysis [4]. Therefore,

$$\frac{amount\ of\ X}{iBAQ\ of\ X} = \frac{amount\ of\ Y}{iBAQ\ of\ Y} \quad (1)$$

where Y is the reference protein and *X* is the protein of interest. Thus, the formula for quantification of surface expression of DR4/5 receptors by comparing their iBAQ scores with the iBAQ score and abundance of TNFR1 is

$$[DR] = \frac{[TNFR1]*iBAQ(DR)}{iBAQ(TNFR1)} \quad (2)$$

The final receptor quantities are summarised in Table A.

Table A: DR4/5 comparative quantification

iBAQ of TNFR1	TNFR1 absolute surface amount, #	iBAQ of DR5	iBAQ of DR4	DR5 absolute surface amount, #	DR4 absolute surface amount, #
4.991199	905	5.109362	4.238999	926.4252	768.6117

Estimation of death ligand content

Considering the molecular weight of the death ligand, the volume of the treatment medium we estimated the number of ligand trimers available per single cell (Table B). The volume of HeLa cell were considered to be fixed and equal to 2.6 pL [5].

Table B: Conversion of death receptor ligand concentration into ligand amount in the proximity volume of a single HeLa cell

Death ligand	Concentration, ng/mL	Concentration, nM	Ligands per each HeLa cell, #	Ligand trimers per cell, #
rhTRAIL	5	0.238	373	124
rhTRAIL	7.5	0.357	559	186
rhTRAIL	50	2.381	3728	1243
rhTRAIL	250	11.905	18640	6213
rhTNFα	25	1.471	2303	768
rhTNFα	10	0.588	921	307

Thus, for example, we are translating the concentration of 10 ng/mL of monomeric (under denaturation conditions) recombinant TNFα with molecular weight of 17kDa into 307 molecules of its trimer available per single HeLa cell. This amount is enough to saturate all trimer TNFR1 complexes that are permanently present at the cellular membrane in the maximum amount of 301 receptor trimers per cell estimated by our experiment. Moreover, this concentration in the treatment medium allows the maintenance of efficient stimulation of all receptors for many more receptor internalisation cycles [6,7].

Estimation of ligand associated death receptor content

Death receptors binding to the dedicated death ligands is at least 3-fold higher [8] than the downstream Casp8 dimerization process [9] and, consequently, Casp8 activation dynamics [10]. This empowers the use of the rapid equilibrium approximation (REA) for the estimation of the ligand-receptor complexes. From REA we derived the relationship between the ligand bound receptors amount $[RL]$ and total number of receptors per cell $[R_{total}]$ (Table A) together with ligand concentration $[L]$ (Table B).

$$\frac{d[RL]}{dt} = k_{on}[R][L] - k_{off}[RL], \qquad where \quad [R] = [R_{total}] - [RL]$$

The stationary equilibrium is:

$$\frac{d[RL]}{dt} = 0, \qquad therefore \qquad [RL] = \frac{[R_{total}]}{\left(\frac{K_d}{[L]} + 1\right)}$$

where K_d is ligand dissociation constant derived from the literature (Table C).

Table C: Ligand to death receptor dissociation constant

Dissociation	Kd value, nM	Reference
rhTRAIL to DR4	8.09	[11]
rhTRAIL to DR5	0.27	[12]
rhTNFα to TNFR1	0.02	[8]

Estimation of ligand associated receptor clustering

The minimal unit of the active receptor-ligand complex is the trimer. Death receptor trimers form higher order structures by clustering together. Both these processes, trimerization and further clustering, are triggered by ligand association [13–15] (S1 Fig). The experimentally measured frequency distribution of the TNFR1 cluster size on the cellular membrane of unstimulated (black line in S1 Fig) and TNFα stimulated HeLa cells derived by Super-resolution PALM microscopy [13] allowed us to calibrate single cell cluster's content. The experimental distribution (pink solid line) was smoothed by splines transformation (red solid line in S1 Fig), segregated by the trimer receptor unit per cluster (red dashed lines in S1 Fig) and quantified (table within S1 Fig).

Measurement and estimation of endogenous protein concentrations

We determined intracellular protein concentrations of FADD, RIP1 in HeLa by (QWB). HeLa cells were harvested and lysed. Western blots were performed using ranges of 10-100 µg of whole cellular protein and 10-2000 fmol of purified recombinant FADD, RIP1.

RIPK1 antibody was purchased from Cell Signaling Technology (Danvers, MA, USA). Caspase 8 antibody was from Alexis Biochemicals (San Diego, CA, USA) and FADD antibody was obtained from BD Transduction Laboratories (Franklin Lakes, NJ, USA). Fluorescent-labelled secondary antibodies were purchased from LI-COR Biosciences (UK).

The fluorescence signals from each western blot were scanned using a Licor Odyssey Infrared Imaging System. The digital densitometry analysis was carried out by quantifying the fluorescent signal intensities of each protein band using the Licor Biosciences software version 3.0.30.

Relating the signal intensities of recombinant proteins to the signal intensities from HeLa lysate, we calculated the amount of the respective protein per HeLa total protein content. Assuming the total protein mass in HeLa cell of 156 pg [16] and the average HeLa volume of 2.6 pL [5], the resulting total protein concentration of 60 µg/µL were used to rescaling the amount of the respective protein per HeLa total protein content to the protein amount per volume of HeLa. Thus, we retrieve the intracellular protein concentration for HeLa cells that we finally converted into number of molecules per single cell (Table D).

Table D: Protein numbers and concentrations

Protein	# molecules per cell in model	Concentration, nM	Reference/Remark
ProCasp8	54801	35.0	[17]
FADD	236574	151.1	experiment
RIP1	1942026	1240.3	experiment
RIP3	100		undetectable, experiment
ProCasp3	187888	120	[18]
ProCasp6	1204	0.77	adjusted
XIAP	98641	63	[18]

ProCasp6 concentration was manually adjusted in simulations of the complete deterministic system which comprised of the averaged stochastic dynamics of Casp8 activation on the RIPoptosome combined with the deterministic ODE system that reproduces Casp8-Casp3-Casp6 feedback (Fig 2C).

The contribution of the FLIP(S) as an inhibition of ProCasp8 dimerization was neglected due to its low concentration compared to ProCasp8 in HeLa cells (Table D). Estimated endogenous level of FLIP(L) in HeLa cells is low [19] therefore its contribution as a dimerization co-partner of ProCasp8 was neglected too in the current modelling setup. RIP3 protein was under the detection limit for the tested dilution range of HeLa lysate in our QWB experiments. A previous study suggests that RIP3 is not expressed endogenously in HeLa cells [20], therefore, we use the arbitrary amount of 100 molecules of RIP3 protein per HeLa cell as the estimate for the low RIP3 content.

Determination of kinetic parameter values

Most of the kinetic parameters in the current modelling set up were taken from the literature (Table E) except the value for Casp8 catalytic efficiency to FRET probe (*keff*) and the rate of Casp3 ubiquitin dependent degradation by XIAP (*kcat*) that, in addition to the ProCasp6 concentration, was adjusted by complete deterministic system (Fig 2C).

Table E: Parameters values

Parameter	Symbol	Value	Unit	Stochastic rate	Unit	Reference/Remark
DEDs-DEDs dissociation constant	Kd	0.175	uM			[21]
DEDs-DEDs dissociation rate	aoff, boff, coff	0.0609	1/min	0.0609	1/min	
DEDs-DEDs association rate	aon, bon, con	0.348	1/(uM*min)	2.22261E-07	1/((#/cell)*min)	[22]
DD-DD domain dissociation rate	doff	0.4651	1/min	0.4651	1/min	[23]
DD-DD dissociation constant	Kd_dd	0.323	uM			[23]
DD-DD domain association rate	don	1.439938	1/(uM*min)	9.1966E-07	1/((#/cell)*min)	
RHIM-RHIM domain dissociation rate	eoff	0.6	1/min	0.6	1/min	[24]
RHIM-RHIM domain association rate	eon	180	1/(uM*min)	0.000114962	1/((#/cell)*min)	[24]
Casp8 dimer dissociation rate	kdoff	0.0257	1/min	0.0257	1/min	[9]
Casp8 dimer dissociation constant	Kd1	3.3	uM			[9]
Casp8 dimerization rate	kdon	0.007788	1/(uM*min)	4.97404E-09	1/((#/cell)*min)	[9,25]
Casp8 catalytic efficiency to ProCasp3	keffc8c3	52.2	1/(uM*min)	3.33391E-05	1/((#/cell)*min)	[26]

Casp3 catalytic efficiency to ProCasp6	keffc3c6	12	1/(uM*min)	7.66416E-06	1/((#/cell)*min)	[27]
Casp6 catalytic efficiency to ProCasp8	keffc6c8	0.0402	1/(uM*min)	2.56749E-08	1/((#/cell)*min)	[28]
Casp3 catalytic efficiency to ProCasp3	keffc3c3	2.4	1/(uM*min)	1.53283E-06	1/((#/cell)*min)	[29]
Casp8 catalytic efficiency to FRET probe	keff	162	1/(uM*min)	0.000103466	1/((#/cell)*min)	Adjusted by model
XIAP binding to Casp3	kxiapon	156	1/(uM*min)	9.96341E-05	1/((#/cell)*min)	[18]
XIAP dissociation from Casp3	kxiapoff	0.144	1/min	0.144	1/min	[18]
Casp3 ubiquitin dependent degradation by XIAP	kcat	1.75<br>0.04	1/min	1.75<br>0.04	1/min	Adjusted by model<br>[30]<br>Initial value

Stochastic DISC/RIPoptosome model

Dynamic assembly of individual RIPoptosome was been simulated stochastically by direct Gillespie stochastic simulation algorithm (SSA) [31,32]. The SSA deals with the discrete reaction species that are the anterior and posterior tails of the individual RIPoptosomes within a single cell. Each tail has a probability either to associate with the compatible protein or to lose the existent proximal protein association. The formation of each RIPoptosome as the linear filament of *n* consequently joined RIPoptosomal proteins summarized by the following reactions:

$$[\text{filament}(n)\_\text{DED domain}] + \text{FADD} \xleftrightarrow[aon/aoff]{} [\text{filament}(n+1)\_\text{DD domain}]\,,$$

$$[\text{filament}(n)\_\text{DED domain}] + \text{ProCasp8} \xleftrightarrow[bon/boff]{} [\text{filament}(n+1)\_\text{DED2 domain}]\,,$$

$$[\text{filament}(n)\_\text{DED domain}] + \text{FLIP(S/L)} \xleftrightarrow[bon/boff]{} [\text{filament}(n+1)\_\text{DED2 domain}]\,,$$

$$[\text{filament}(n)\_\text{DED domain}] + \text{Casp8 DEDs} \xleftrightarrow[bon/boff]{} [\text{filament}(n+1)\_\text{DED2 domain}]\,,$$

$$[\text{filament}(n)\_\text{DED2 domain}] + \text{ProCasp8} \xleftrightarrow[con/coff]{} [\text{filament}(n+1)\_\text{DED2 domain}]\,,$$

$$[\text{filament}(n)\_\text{DED2 domain}] + \text{FLIP(S/L)} \xleftrightarrow[con/coff]{} [\text{filament}(n+1)\_\text{DED2 domain}]\,,$$

$$[\text{filament}(n)\_\text{DED2 domain}] + \text{Casp8 DEDs} \xleftrightarrow[con/coff]{} [\text{filament}(n+1)\_\text{DED2 domain}]\,,$$

$$[\text{filament}(n)\_\text{DD domain}] + \text{FADD} \xleftrightarrow[don/doff]{} [\text{filament}(n+1)\_\text{DED domain}]\,,$$

$$[\text{filament}(n)\_\text{DD domain}] + \text{RIP1} \xleftrightarrow[don/doff]{} [\text{filament}(n+1)\_\text{RHIM domain}]\,,$$

$$[\text{filament}(n)\_\text{RHIM domain}] + \text{RIP1} \xleftrightarrow[eon/eoff]{} [\text{filament}(n+1)\_\text{DD domain}]\,,$$

$$[\text{filament}(n)\_\text{RHIM domain}] + \text{RIP3} \xleftrightarrow[eon/eoff]{} [\text{filament}(n+1)\_\text{RHIM domain}]\,.$$

The propensity of each reaction at the each iteration step was calculated from the dedicated association/dissociation rate constant shown in Table E and the current amount of compatible

reaction species. The sequential association of two molecules of ProCasp8 with one of the filaments tails is taken to coincide with ProCasp8 self-cleavage. This results in the formation of two Casp8 DEDs that remain associated with the filament and creation of a single active dimeric Casp8 (Casp8*) which dissociates from the filament. The same Casp8 activation is considered in the case of receptor clustering scenario when simultaneous binding of two molecules of ProCasp8 take place within one cluster. Anterior and posterior sides of the filaments within the cluster are considered independently.

Complete deterministic model

To identify the values for free parameters, Casp8 catalytic efficiency to FRET probe (*keff*), the rate of Casp3 ubiquitin dependent degradation (*kcat*) and ProCasp6 concentration, we formalized the deterministic model of the whole pre-MOMP apoptotic network. The reactions incorporated in the model are following:

$$\mathrm{ProCasp8} + \mathrm{ProCasp8} \xrightarrow[kact]{} \mathrm{Casp8}^* \,,$$

$$\mathrm{Cs8}^* + \mathrm{Cs8}^* \xleftrightarrow[kdon/kdoff]{} \mathrm{Casp8}^* \,,$$

$$\mathrm{Casp8}^* + \mathrm{ProCasp3} \xrightarrow[keffc8c3]{} \mathrm{Casp8}^* + \mathrm{Casp3}^* \,,$$

$$\mathrm{Casp8}^* + \mathrm{FRETprobe} \xrightarrow[keff]{} \mathrm{Casp8}^* + \mathrm{FRETcleaved} \,,$$

$$2\mathrm{Casp3}^* + \mathrm{ProCasp3} \xrightarrow[keffc3c3]{} 2\mathrm{Casp3}^* + \mathrm{Casp3}^* \,,$$

$$2\mathrm{Casp3}^* + \mathrm{ProCasp6} \xrightarrow[keffc3c6]{} 2\mathrm{Casp3}^* + \mathrm{Casp6}^* \,,$$

$$2\mathrm{Casp6}^* + \mathrm{ProCasp8} \xrightarrow[keffc6c8]{} 2\mathrm{Casp6}^* + \mathrm{Cs8}^* \,,$$

$$\mathrm{Casp3}^* + \mathrm{XIAP} \xleftrightarrow[kxiapon/kxiapoff]{} \mathrm{Casp3}^*\!:\mathrm{XIAP} \,,$$

$$\mathrm{Casp3}^*\!:\mathrm{XIAP} \xrightarrow[kcat]{} \mathrm{Casp3}^*\mathrm{ub} \,.$$

Taking into account the law of total mass conservation of each protein, the model reactions translated into a system of ordinary differential equations (ODE), describing the time evolution of seven core variables (Table F) constituting the network (Fig 2C).

<u>**Table F:** ODE variables</u>

**Variable**	**Symbol**
Dimer of active Caspase 8 (Casp8*)	$y(1)$
Monomer of active Caspase 3 (Casp3*)	$y(2)$
Monomer of active Caspase 6 (Casp6*)	$y(3)$
Monomer of active Caspase 8 (Cs8*)	$y(4)$
Monomeric active Caspase 3 in the complex with XIAP (Casp3*:XIAP)	$y(5)$
Ubiquitinated monomeric Caspase 3 (Casp3*ub)	$y(6)$
Cleaved FRET probe (FRETcleaved)	$y(7)$

We have implemented the deterministic rate constant for the RIPoptosome based Casp8 activation dynamics (*kact*). For that we manually adjusted *kact* to reproduce the population average dynamics of stochastic RIPoptosome based Casp8 activation alone over the population of 100 cells averaging the time traces for the response to 5, 7.5, 50, 250 ng/mL of the death ligand. Thus, the final ODE system is expressed as following:

$$\frac{dy(1)}{dt} = kact\,\left(ProCas8 - 2y(1) - y(4)\right)^2 + kdon\,y(4)^2 - kdoff\,y(1)$$

$$\frac{dy(2)}{dt} = (keffc8c3\,y(1) + keffc3c3\,y(2)^2)\left(ProCas3 - y(2) - y(5) - y(6)\right) - kxiapon\,y(2)\,\left(XIAP - y(5)\right) + kxiapoff\,y(5)$$

$$\frac{dy(3)}{dt} = keffc3c6\,y(2)^2\,(ProCas6 - y(3))$$

$$\frac{dy(4)}{dt} = keffc6c8\,y(3)^2\left(ProCas8 - 2y(1) - y(4)\right) + 2\,kdoff\,y(1) - 2\,kdon\,y(4)^2$$

$$\frac{dy(5)}{dt} = kxiapon\,y(2)\left(XIAP - y(5)\right) - (kxiapoff + kcat)\,y(5)$$

$$\frac{dy(6)}{dt} = kcat\,y(5)$$

$$\frac{dy(7)}{dt} = keff\,y(1)(1 - y(7))$$

With this model we manually adjusted *keff*, *kcat* and *ProCasp6* parameters. The satisfying parameter set reproduces the average dependence of the experimentally observed HeLa cell death delays. We have considered the mean of the cell death delay distributions quantified in the experiments [33] for of FRET probe cleavage associated with the MOMP. The final parameter values are shown in the table (Table E).

Semi-stochastic hybrid model implementation

To merge the dynamics of the discrete process of Casp8 production on the RIPoptosome and continuous system of downstream effector Caspases feedback loop we combined the Monte Carlo algorithm of discrete simulation with the continuous time-step integration of ODE system. Considering a very low population number of RIPoptosomes origins per cell compared to other reactive species in the network, the system can be partitioned based on species population number [34,35]. Thus we partition the system into fixed set of slow reactions that are the origin based assembly of RIPoptosome and fixed set of the fast reactions that are the downstream caspases cascade. According to the earlier develop algorithm for stochastic-deterministic coupling [35,36] we simulate a slow reaction group by direct Gillespie SSA [31,32] and subset of the fast reactions with ODE integration approach. We adapted earlier published algorithm of direct stochastic-deterministic variables coupling [37] and implemented our iteration pipeline of trade-off between direct SSA and ODE integration in the MATLAB 2017b environment. To verify the accuracy of the hybrid method in

our case we compared it with the fully stochastic method. Figure S5 demonstrates that two approaches are quantitatively and qualitatively in agreement.

The pipeline

Set time $t = 0$ and set discrete and continuous variables initial conditions $y(0) = initial\ values$. Calculate the propensity for each discrete reaction from the set of discrete reactions.

While *t* is less than the simulation duration

1) From the propensities of the discrete reactions, select a time step size τ and the reaction to happen over τ period according to Gillespie SSA.
2) Update initial conditions $y(t)$ for the deterministic system according to output of step 1.
3) Integrate **ODE1 system** of continuous reactions over the time interval $[t, t + \tau]$ and get $y(t + \tau)$.
4) Update the propensities of the discrete reactions according to the $y(t + \tau)$ from step 3.
5) Set t to the $t + \tau$

End

ODE1 system

We present below the system of deterministic ODEs adapted for our hybrid model simulations where amount of active dimers of Casp8, $y(1)$, and the amount of ProCasp8 molecules recruited to RIPoptosome, $ProCasp8bound$, are the bridging species between the stochastic and deterministic routine:

$$\frac{dy(1)}{dt} = kdon\, y(4)^2 - kdoff\, y(1)$$

$$\frac{dy(2)}{dt} = (keffc8c3\, y(1) + keffc3c3\, y(2)^2)\big(ProCas3 - y(2) - y(5) - y(6)\big) - kxiapon\, y(2)\,\big(XIAP - y(5)\big) + kxiapoff\, y(5)$$

$$\frac{dy(3)}{dt} = keffc3c6\, y(2)^2\,(ProCas6 - y(3))$$

$$\frac{dy(4)}{dt} = keffc6c8\, y(3)^2(ProCas8 - 2y(1) - y(4) - ProCas8bound) + 2\,kdoff\, y(1) - 2\,kdon\, y(4)^2$$

$$\frac{dy(5)}{dt} = kxiapon\, y(2)\big(XIAP - y(5)\big) - (kxiapoff + kcat)\, y(5)$$

$$\frac{dy(6)}{dt} = kcat\, y(5)$$

$$\frac{dy(7)}{dt} = keff\, y(1)(1 - y(7))$$

Coefficient of variation

The coefficient of variation (CV) was used as the relative measure of noise strength:

$$CV = \frac{Standart\ deviation\ of\ FRET\ signal}{Mean\ of\ FRET\ signal}$$

For single cell dynamic ramp noise the CV was calculated through the time evolution from the moment of dose introduction till the last 30 min before the cell death threshold.

Bimodality test

In order to verify that the low dose DL with DR clustering scenario exhibited bimodality (Fig 6A), we performed a statistical test outlined in [38] and implemented in the R package ‘multimode'. This method utilises an excess mass statistic with bootstrap calibration. We found that in this case the true number of modes is greater than 1 with p-value < 2.2e-16 but is greater than 2 with p-value = 0.8. Therefore the number of modes in this case is exactly 2. Though, for the low dose DL with the disrupted clustering scenario (Fig 6C) we found the true number of modes to be greater than 1 with p-value = 0.87, therefore in this case the distribution is unimodal.

References

1. Schwanhäusser B, Busse D, Li N, Dittmar G, Schuchhardt J, Wolf J, et al. Global quantification of mammalian gene expression control. Nature. 2011;473: 337–342. doi:10.1038/nature10098

2. Mohr A, Yu R, Zwacka RM. TRAIL-receptor preferences in pancreatic cancer cells revisited: Both TRAIL-R1 and TRAIL-R2 have a licence to kill. BMC Cancer. 2015;15: 494. doi:10.1186/s12885-015-1508-2

3. Geiger T, Wehner A, Schaab C, Cox J, Mann M. Comparative proteomic analysis of eleven common cell lines reveals ubiquitous but varying expression of most proteins. Mol Cell Proteomics. 2012;11: M111.014050. doi:10.1074/mcp.M111.014050

4. Ahrné E, Molzahn L, Glatter T, Schmidt A. Critical assessment of proteome-wide label-free absolute abundance estimation strategies. Proteomics. 2013;13: 2567–2578. doi:10.1002/pmic.201300135

5. Zhao L, Kroenke CD, Song J, Piwnica-Worms D, Ackerman JJH, Neil JJ. Intracellular water-specific MR of microbead-adherent cells: the HeLa cell intracellular water exchange lifetime. NMR Biomed. 2008;21: 159–164. doi:10.1002/nbm.1173

6. Fallahi-Sichani M, El-Kebir M, Marino S, Kirschner DE, Linderman JJ. Multiscale computational modeling reveals a critical role for TNF-$α$ receptor 1 dynamics in tuberculosis granuloma formation. J Immunol. Am Assoc Immnol; 2011;186: 3472–3483.

7. Heidbreder M, Zander C, Malkusch S, Widera D, Kaltschmidt B, Kaltschmidt C, et al. TNF-α influences the lateral dynamics of TNF receptor I in living cells. Biochim Biophys Acta. 2012;1823: 1984–1989. doi:10.1016/j.bbamcr.2012.06.026

8. Grell M, Wajant H, Zimmermann G, Scheurich P. The type 1 receptor (CD120a) is the high-affinity receptor for soluble tumor necrosis factor. Proc Natl Acad Sci U S A. 1998;95: 570–575.

9. Pop C, Fitzgerald P, Green DR, Salvesen GS. Role of proteolysis in caspase-8 activation and stabilization. Biochemistry. ACS Publications; 2007;46: 4398–4407.

10. Hellwig CT, Kohler BF, Lehtivarjo A-K, Dussmann H, Courtney MJ, Prehn JHM, et al. Real time

analysis of tumor necrosis factor-related apoptosis-inducing ligand/cycloheximide-induced caspase activities during apoptosis initiation. J Biol Chem. 2008;283: 21676–21685. doi:10.1074/jbc.M802889200

11. Dufour F, Rattier T, Shirley S, Picarda G, Constantinescu AA, Morlé A, et al. N-glycosylation of mouse TRAIL-R and human TRAIL-R1 enhances TRAIL-induced death. Cell Death Differ. 2017;24: 500–510. doi:10.1038/cdd.2016.150

12. Pavet V, Beyrath J, Pardin C, Morizot A, Lechner M-C, Briand J-P, et al. Multivalent DR5 peptides activate the TRAIL death pathway and exert tumoricidal activity. Cancer Res. 2010;70: 1101–1110. doi:10.1158/0008-5472.CAN-09-2889

13. Fricke F, Malkusch S, Wangorsch G, Greiner JF, Kaltschmidt B, Kaltschmidt C, et al. Quantitative single-molecule localization microscopy combined with rule-based modeling reveals ligand-induced TNF-R1 reorganization toward higher-order oligomers. Histochem Cell Biol. Springer; 2014;142: 91–101.

14. Graves JD, Kordich JJ, Huang T-H, Piasecki J, Bush TL, Sullivan T, et al. Apo2L/TRAIL and the death receptor 5 agonist antibody AMG 655 cooperate to promote receptor clustering and antitumor activity. Cancer Cell. 2014;26: 177–189. doi:10.1016/j.ccr.2014.04.028

15. Valley CC, Lewis AK, Mudaliar DJ, Perlmutter JD, Braun AR, Karim CB, et al. Tumor necrosis factor-related apoptosis-inducing ligand (TRAIL) induces death receptor 5 networks that are highly organized. J Biol Chem. 2012;287: 21265–21278. doi:10.1074/jbc.M111.306480

16. Finka A, Goloubinoff P. Proteomic data from human cell cultures refine mechanisms of chaperone-mediated protein homeostasis. Cell Stress Chaperones. 2013;18: 591–605. doi:10.1007/s12192-013-0413-3

17. Eissing T, Conzelmann H, Gilles ED, Allgöwer F, Bullinger E, Scheurich P. Bistability analyses of a caspase activation model for receptor-induced apoptosis. J Biol Chem. 2004;279: 36892–36897. doi:10.1074/jbc.M404893200

18. Rehm M, Huber HJ, Dussmann H, Prehn JHM. Systems analysis of effector caspase activation and its control by X-linked inhibitor of apoptosis protein. EMBO J. 2006;25: 4338–4349. doi:10.1038/sj.emboj.7601295

19. Albeck JG, Burke JM, Spencer SL, Lauffenburger DA, Sorger PK. Modeling a snap-action, variable-delay switch controlling extrinsic cell death. PLoS Biol. 2008;6: 2831–2852. doi:10.1371/journal.pbio.0060299

20. Li J, McQuade T, Siemer AB, Napetschnig J, Moriwaki K, Hsiao Y-S, et al. The RIP1/RIP3 necrosome forms a functional amyloid signaling complex required for programmed necrosis. Cell. 2012;150: 339–350. doi:10.1016/j.cell.2012.06.019

21. Fu T-M, Li Y, Lu A, Li Z, Vajjhala PR, Cruz AC, et al. Cryo-EM Structure of Caspase-8 Tandem DED Filament Reveals Assembly and Regulation Mechanisms of the Death-Inducing Signaling Complex. Mol Cell. 2016;64: 236–250. doi:10.1016/j.molcel.2016.09.009

22. Ma C, MacKenzie SH, Clark AC. Redesigning the procaspase-8 dimer interface for improved dimerization. Protein Sci. 2014;23: 442–453. doi:10.1002/pro.2426

23. Park Y-H, Jeong MS, Park HH, Jang SB. Formation of the death domain complex between FADD and RIP1 proteins in vitro. Biochim Biophys Acta. 2013;1834: 292–300. doi:10.1016/j.bbapap.2012.08.013

24. Cohen SIA, Linse S, Luheshi LM, Hellstrand E, White DA, Rajah L, et al. Proliferation of amyloid-β42 aggregates occurs through a secondary nucleation mechanism. Proc Natl Acad Sci U S A. 2013;110: 9758–9763. doi:10.1073/pnas.1218402110

25. Würstle ML, Laussmann MA, Rehm M. The caspase-8 dimerization/dissociation balance is a highly potent regulator of caspase-8, -3, -6 signaling. J Biol Chem. 2010;285: 33209–33218. doi:10.1074/jbc.M110.113860

26. Stennicke HR, Jürgensmeier JM, Shin H, Deveraux Q, Wolf BB, Yang X, et al. Pro-caspase-3 is a major physiologic target of caspase-8. J Biol Chem. 1998;273: 27084–27090.

27. Stennicke HR, Renatus M, Meldal M, Salvesen GS. Internally quenched fluorescent peptide substrates disclose the subsite preferences of human caspases 1, 3, 6, 7 and 8. Biochem J. 2000;350 Pt 2: 563–568.

28. Julien O, Zhuang M, Wiita AP, O'Donoghue AJ, Knudsen GM, Craik CS, et al. Quantitative MS-based enzymology of caspases reveals distinct protein substrate specificities, hierarchies, and cellular roles. Proc Natl Acad Sci U S A. 2016;113: E2001--E2010. doi:10.1073/pnas.1524900113

29. Zou H, Yang R, Hao J, Wang J, Sun C, Fesik SW, et al. Regulation of the Apaf-1/caspase-9 apoptosome by caspase-3 and XIAP. J Biol Chem. 2003;278: 8091–8098. doi:10.1074/jbc.M204783200

30. Van Voorhis VA, Morgan DO. Activation of the APC/C ubiquitin ligase by enhanced E2 efficiency. Curr Biol. 2014;24: 1556–1562. doi:10.1016/j.cub.2014.05.052

31. Gillespie DT. Exact stochastic simulation of coupled chemical reactions. J Phys Chem. ACS Publications; 1977;81: 2340–2361.

32. Gillespie DT. A general method for numerically simulating the stochastic time evolution of coupled chemical reactions. J Comput Phys. Elsevier; 1976;22: 403–434.

33. Roux J, Hafner M, Bandara S, Sims JJ, Hudson H, Chai D, et al. Fractional killing arises from cell-to-cell variability in overcoming a caspase activity threshold. Mol Syst Biol. 2015;11: 803. doi:10.15252/msb.20145584

34. Cao Y, Gillespie DT, Petzold LR. Avoiding negative populations in explicit Poisson tau-leaping. J Chem Phys. 2005;123: 54104. doi:10.1063/1.1992473

35. Haseltine EL, Rawlings JB. Approximate simulation of coupled fast and slow reactions for stochastic chemical kinetics. J Chem Phys. AIP; 2002;117: 6959–6969.

36. Liu Z, Pu Y, Li F, Shaffer CA, Hoops S, Tyson JJ, et al. Hybrid modeling and simulation of stochastic effects on progression through the eukaryotic cell cycle. J Chem Phys. 2012;136: 34105. doi:10.1063/1.3677190

37. Kiehl TR, Mattheyses RM, Simmons MK. Hybrid simulation of cellular behavior. Bioinformatics. 2004;20: 316–322. doi:10.1093/bioinformatics/btg409

38. Ameijeiras-Alonso J, Crujeiras RM, Rodr\'\iguez-Casal A. Mode testing, critical bandwidth and excess mass. Test. Springer; 2018; 1–20.
